# Dihydroxyphenyl- and Heteroaromatic-Based Thienopyrimidinones to Tackle HIV-1 LEDGF/p75-Dependent IN Activity

**DOI:** 10.3390/molecules28186700

**Published:** 2023-09-19

**Authors:** Graziella Tocco, Serena Canton, Antonio Laus, Pierluigi Caboni, Stuart F. J. Le Grice, Enzo Tramontano, Francesca Esposito

**Affiliations:** 1 Department of Life and Environmental Sciences, University of Cagliari, Cittadella Universitaria di Monserrato, 09042 Monserrato, Italy; serena3canton@gmail.com (S.C.); antolaus@unimore.it (A.L.); caboni@unica.it (P.C.); tramon@unica.it (E.T.); francescaesposito@unica.it (F.E.); 2 Center for Cancer Research, National Cancer Institute, Frederick, MD 21702-1201, USA; garysbruv@gmail.com

**Keywords:** HIV-1 integrase (IN), allosteric inhibitors, thienopyrimidinones, LEDGF/p75-dependent IN activity

## Abstract

The spread of Human Immunodeficiency Virus (HIV) still represents a global public health issue of major concern, and would benefit from unveiling unique viral features as targets for drug design. In this respect, HIV-1 integrase (IN), due to the absence of homologs in human cells, is a popular target for the synthesis of novel selective compounds. Moreover, as drug-resistant viral strains are rapidly evolving, the development of novel allosteric inhibitors is acutely required. Recently, we have observed that Kuwanon-L, quinazolinones and thienopyrimidinones containing at least one polyphenol unit, effectively inhibited HIV-1 IN activity. Thus, in the present research, novel dihydroxyphenyl-based thienopyrimidinone derivatives were investigated for their LEDGF/p75-dependent IN inhibitory activity. Our findings indicated a close correlation between the position of the OH group on the phenyl moiety and IN inhibitory activity of these compounds. As catechol may be involved in cytotoxicity, its replacement by other aromatic scaffolds was also exploited. As a result, compounds **21**–**23**, **25** and **26** with enhanced IN inhibitory activity provided good lead candidates, with **25** being the most selective for IN. Lastly, UV spectrometric experiments suggested a plausible allosteric mode of action, as none of the thienopirimidinones showed Mg^2+^ chelation properties otherwise typical of IN strand transfer inhibitors (INSTIs).

## 1. Introduction

According to the World Health Organization, HIV transmission still represents an important worldwide public health problem, with around 1.3 million new infections worldwide by 2022 [1] mainly related to HIV-1, the agent responsible for the acquired immunodeficiency syndrome (AIDS).

Although many therapeutic strategies have been developed to strengthen the cART arsenal, the evolution of drug-resistant viral strains remains a serious issue. Thus, identifying novel viral factors as targets for drug design remains a priority. In this regard, HIV-1 integrase (IN) is an attractive target for new systematic drug discovery efforts, especially because of the absence of a cellular homolog that may result in a reduced occurrence of adverse effects [2].

IN is a multimeric enzyme and, together with reverse transcriptase (RT) and protease, is one of the three viral enzymes necessary for HIV-1 replication. It catalyzes the incorporation of double-stranded proviral DNA into the host cell genome [3] and comprises three domains: the N-terminal region, the central core domain containing the catalytic triad DDE that chelates two Mg^2+^ ions, and the C-terminal region [4]. In particular, the integration process requires the following two steps: (1) in the cytosol, the IN dimer catalyzes cleavage of a guanine–thymine (GT) dinucleotide from the 3′-terminus of each viral DNA end (*3′-processing*) and (2) in the nucleus, tetrameric IN mediates integration of recessed viral DNA ends into the cellular DNA via a concerted hydroxy-mediated nucleophilic reaction (*DNA strand transfer*).

The advent of a convenient in vitro functional assay based on recombinant IN [5] prompted drug discovery efforts to inhibit its DNA strand transfer activity by targeting the active site. As a result, strand transfer inhibitors (INSTIs) Raltegravir (RAL) [6,7], Elvitegravir (EVG) [8], Dolutegravir (DTG) [9], Cabotegravir [10] and Bictegravir [11] have been developed and approved for clinical use. Regrettably, the rapid emergence of viral variants displaying cross-resistance to the FDA-licensed INSTIs highlights the need to develop inhibitors with unique modes of action.

In this regard, the identification [12] and confirmation [13,14,15,16,17,18] of Lens Epithelium-Derived Growth Factor (LEDGF/p75) as an HIV-1 IN binding partner has serendipitously opened up a new route in antiviral drug design, and a series of novel allosteric inhibitors of the LEDGF/p75 binding site (LEDGINs) preventing viral replication by inducing inactive or aberrant IN oligomerization have been reported [19,20,21,22,23,24]. However, the development of small molecules (*allosteric inhibitors*) that access IN elsewhere, and which dramatically hamper activity while still retaining potency against INSTIs and LEDGIN-resistant viral strains, is urgently warranted.

**Figure 1:**
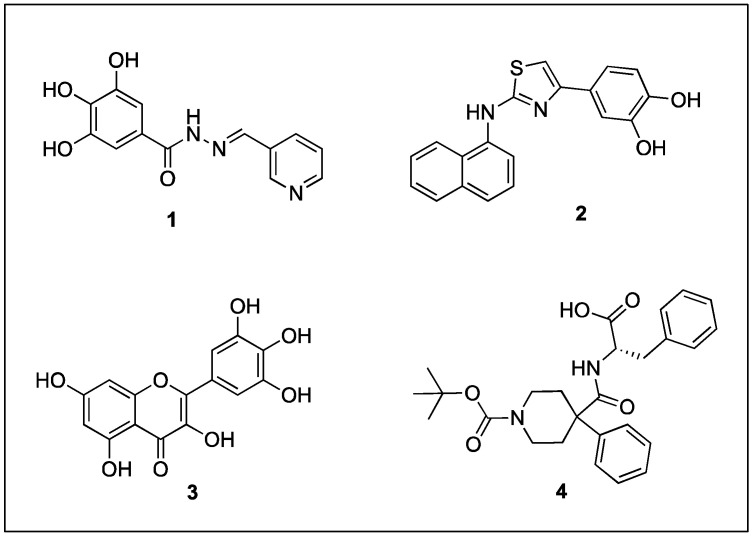
Examples of chemical scaffolds obtained from high throughput screening approach.

Interestingly, a recent high throughput screening study provided a series of different promising hit to lead IN-inhibitor scaffolds from which the (1-(*tert*-butoxycarbonyl)-4-phenylpiperidine-4-carbonyl)phenylalanine **4** was selected and used for further optimization [25] (Figure 1). In addition, the inhibitory activity of various natural flavonols containing polyphenol moieties, e.g., angoletin, quercetin, myricetin and kaempferol has been previously reported [26,27,28].

**Figure 2:**
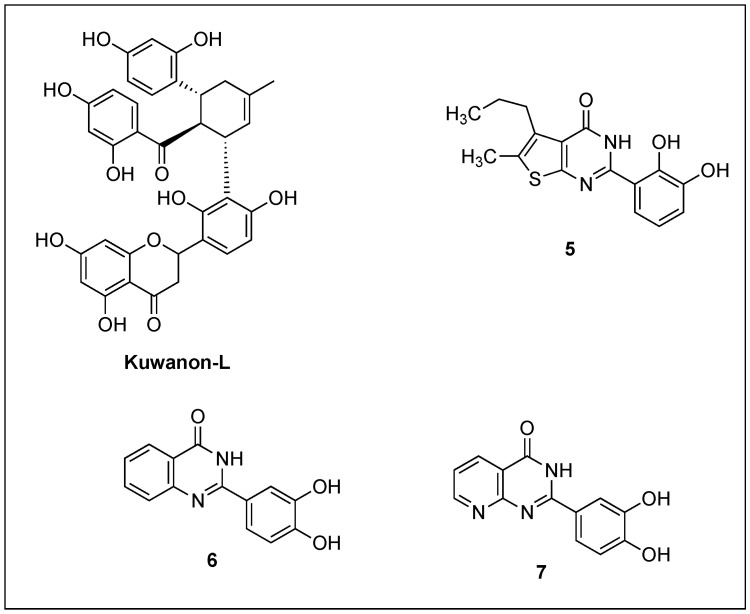
Chemical structure of Kuwanon-L and of most active thienopyrimidinones and quinazolinones.

The increasing interest in novel allosteric IN binding sites prompted some of us to investigate the recently discovered sucrose binding pocket, identifying Kuwanon-L (Figure 2) as a novel allosteric inhibitor. In silico investigation supported the biological findings, also shedding light on the possible binding mode of the polyphenolic scaffold within the sucrose binding pocket [29].

Recently, we also reported on the ability of some 3,4-catechol-thienopyrimidinone [30,31] and quinazolinone derivatives [32], originally designed to allosterically inhibit the HIV-1 RT-associated Ribonuclease H (RNase H) function, to hamper LEDGF/p75-dependent IN activity. Interestingly, with regard to thienopyrimidinones, we observed that the 2-(2,3-dihydroxyphenyl)-6-methyl-5-propylthieno[2,3-*d*]pyrimidin-4(3*H*)-one (**5**), bearing a 2,3-catecol moiety, displayed the best IN inhibitory activity [30]. Figure 2 shows the most active compounds.

The thienopyrimidinone framework is widely documented in many biologically active compounds such as anti-tumor [33], anti-malarial [34,35], anti-microbial and anti-fungal [36], anti-inflammatory [37] and anti-tubercular [38] agents.

Thus, in the present paper, we investigated the LEDGF/p75-dependent IN activity of a series of dihydroxyphenyl-thienopyrimidinones using the strand transfer inhibitor Raltegravir as a positive control. Our results demonstrated a strict relationship between the position of the OH groups on the phenyl moiety and their IN inhibitory activity. Moreover, UV spectrometric experiments demonstrated that all of the thienopirimidinones were unable to chelate Mg^2+^, thereby suggesting a possible allosteric mode of action. As the catechol scaffold—although present in many biologically active compounds—may be associated with cytotoxicity [39,40], we also synthesized and explored the inhibitory activity of differently substituted thienopyrimidinones. They were also examined for their RT-RNase H activity using 2-(3,4-dihydroxyphenyl)-5,6-dimethylthieno[2,3-*d*]pyrimidin-4(3*H*)-one (GZ 510) [30] as a positive control.

## 2. Results

**Figure 3:**
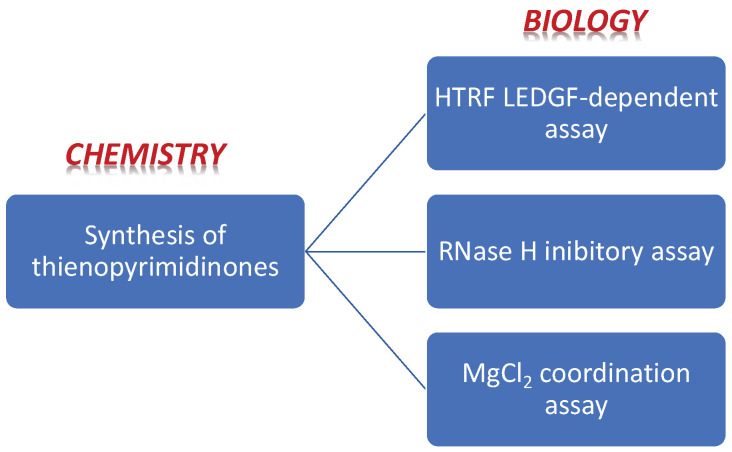
Methodology flowchart.

Methods of investigation are shown in Figure 3.

### 2.1. Chemistry

**Figure 4:**
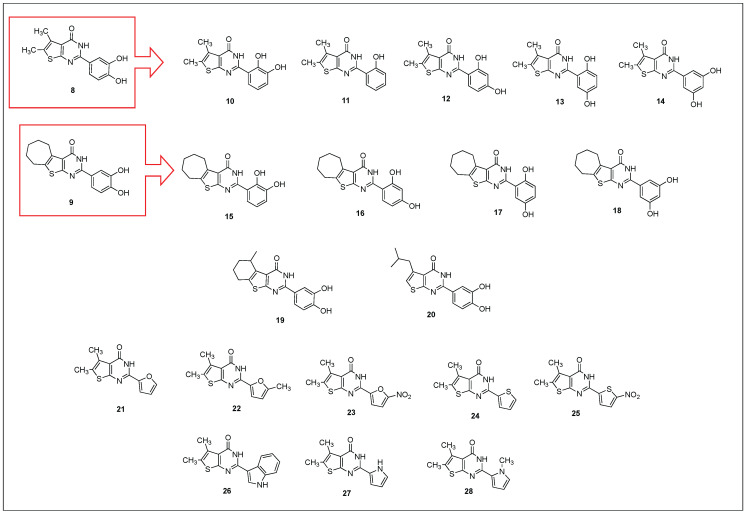
Chemical structures of thienopyrimidinones tested for their effect on the IN LEDGF/p75-dependent activity.

Figure 4 presents thienopyrimidinones investigated in the present research.

**Scheme 1:**
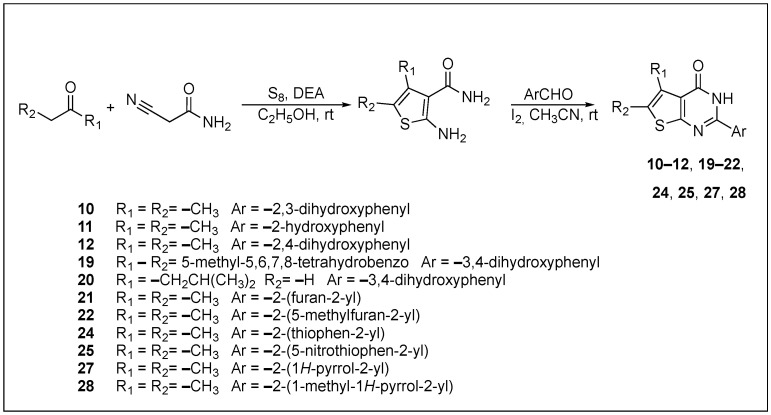
Synthetic pathway for compounds **10**–**12**, **19**–**22**, **24**, **25**, **27** and **28**.

As reported in Scheme 1, the novel thienopyrimidinones were synthesized according to our previously reported procedure for preparation of compounds **8**, **9**, **13**, **14**, **15**–**18**, **23** and **26** [30]. In brief, a Gewald reaction between cyanoacetamide, the appropriate carbonyl derivative, and elemental sulfur in the presence of diethylamine (DEA) as a base, was firstly carried out to afford the correspondent 2-amino-4,5-disubstituted-thiophene-3-carboxamide. Subsequently, the thiophene intermediate reacted via direct oxidative condensation with the proper aldehyde in the presence of molecular iodine, to give rise to the final products **10**–**12**, **19**–**22**, **24**, **25**, **27** and **28**.

### 2.2. Biology

All of the thienopyrimidinones were tested for their ability to affect the IN-LEDGF/p75 dependent activity.

From the analysis of the first series of compounds, it was immediately evident how the relative OH position on the aromatic core influenced the inhibitors’ IC_50_ (Table 1). Remarkably, moving the OH from 4- to 2-position seemed determinant for the improved activity of compound **10**. Other possible combinations were explored, but the distancing of the second OH group negatively affected the inhibitory activity of compounds **12** and **13**. The 3,5-dihydroxyphenyl core appeared to be the best combination, and 2-(3,5-dihydroxyphenyl)-5,6-dimethylthieno[2,3-*d*]pyrimidin-4(3*H*)-one **14** displayed the best IC_50_ value.

**Figure 5:**
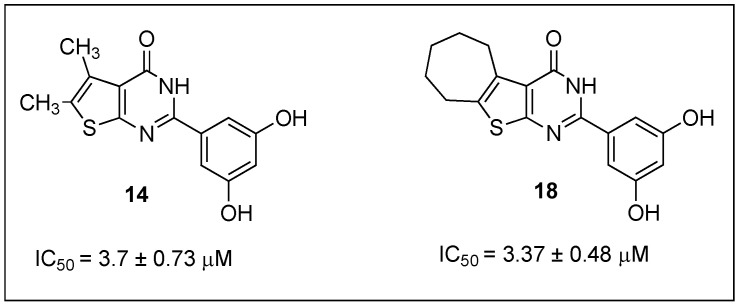
Chemical structure of dihydroxyphenyl thienopyrimidinones with the best IN inhibitory activity.

Introducing more bulky substituents on the thiophene scaffold showed a different trend. In this case, distancing of the second OH group from the 2-hydroxy moiety improved inhibition of the IN LEDGF/p75-dependent activity, as demonstrated by the 3,5,6,7,8,9-hexahydro-4*H*-cyclohepta[4,5]thieno[2,3-*d*]pyrimidin-4-ones **16** and **17**. Once again, the 3,5-dihydroxyphenyl unit proved the optimal core, with compound **18** being the best inhibitor of both series of thienopyrimidinones (Table 2 and Figure 5).

**Figure 6:**
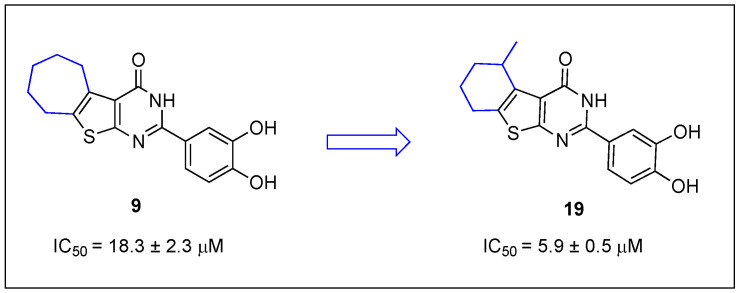
Effect of the replacement of cyclohepthyl ring with a branched cyclohexane on IN inhibitory activity.

These results prompted us to explore substitution on the thiophene ring, and the most interesting compounds are reported in Table 3. Replacing the cyclohepthyl ring of **9** with a branched cyclohexane dramatically improved the inhibitory activity of compound **19** (Figure 6). Similarly, substitution of methyl groups of **8** with the bulkier and more flexible isobutyl moiety generated the more active thienopyrimidinone **20** (Table 3).

**Figure 7:**
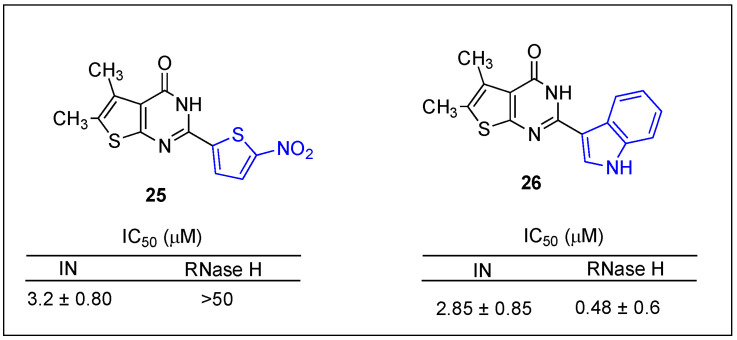
Chemical structure of the most promising heteroaromatic-based thienopyrimidinones.

As the presence of dihydroxyphenyls may induce cytotoxicity [39,40], we addressed the possibility of replacing the dihydroxyphenyl moiety with other aromatic scaffolds, testing the inhibitory activity on both IN and RNase H functions (Table 4). Compounds **21**–**23** and **26** inhibited both IN and RNase-H activity, with **22** and **23** being more selective against IN, while introducing a -NO_2_ group on the thiophene core of **24** totally reversed its activity, making compound **25** completely IN selective (Table 4 and Figure 7). Intriguingly, both pyrrole compounds **27** and **28** were completely IN inactive, while the indole derivative **26** showed a good activity on both IN and RNase-H function (Table 4 and Figure 7).

Finally, to investigate the possible IN inhibition mechanism, active thienopyrimidinones were tested for their metal-chelating properties regarding Mg^2+^ ions implicated as cofactors in the mode of action of INSTIs [41]. Interestingly, although catechol-based compounds are especially notorious for their metal complexing properties, our UV spectrometric experiments excluded the participation of Mg^2+^ cations in their mode of action (see Supplementary Material).

## 3. Discussion

A series of thienopyrimidinones have been tested for their in vitro inhibitory activity on IN catalytic function. We chose the LEDGF/p75-dependent integration assay, as it is a versatile tool for detecting alteration of various IN functions such as multimerization, LEDGF/p75 binding and catalytic activity [25].

**Figure 8:**
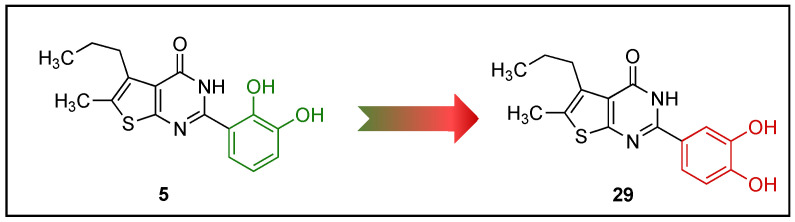
Chemical structure of compounds **5** and **29**.

Structurally, a 3,4-catechol unit at the C-2 position of the thienopyrimidinone scaffold, regardless of any other substitution on the thiophene core, was a key factor in consistently fostering HIV-1 RT-RNase H but not IN activity [30]. As a matter of fact, in our first experiment we observed that switching from 2,3- to 3,4- catechol turned **5** in the IN inactive compound **29** (IC_50_ > 50 mM) (Figure 8).

These observations prompted us to further investigate the relationship between the position of OH substitutions and IN LEDGF/p75-dependent activity.

**Figure 9:**
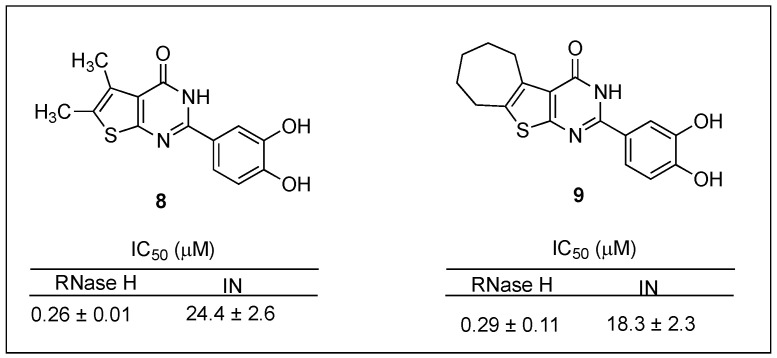
HIV-1 RT-RNase H and IN LEDGF/p75-dependent activity of compounds **8** and **9**.

Firstly, compounds **8** and **9** were selected as a baseline, since they were the most active against RNAse H and very poorly active against IN (Figure 9), and several derivatives exploring all the possible OH combinations were investigated.

The results shown in Table 1 and Table 2 are suggestive of a cooperative relationship between the OH relative position and the thiophene substitution. In particular, as also evidenced in Table 3, bulkier and branched alkyl substitution in 5,6 positions may enhance IN inhibitory activity.

Moreover, compound **11**, bearing only a 2-hydrophenyl core, was slightly more active than **8**, hypothesizing a cooperative intramolecular hydrogen bonding between amide and hydroxy moiety.

Although the catechol unit is present in a wide variety of biologically active compounds, there is no doubt that its redox reactivity may be related to cytotoxicity [39,40]. Thus, a series of thienopyrimidinones supporting different heteroaromatic cores have been investigated (Table 4). Our first candidate, the electron-rich furan ring often used as a bioisosteres of benzene core [42] provided a valid alternative, leading to compound **21** (RNase H IC_50_ of 2.5 ± 0.1 and IN IC_50_ of 5.75 ± 0.85). Introducing an electron-donating methyl group in 5-position resulted in a reduced activity of **22**, even showing a higher IN selectivity. Conversely, the presence of the strong electron withdrawing unit NO_2_, also able to act as an H-bond acceptor as in compound **23**, was a more successful substitution for IN activity while still maintaining selectivity against RNase H function. The presence of the more aromatic thiophene ring led to the inactive compound **24**. As already observed with **23**, once again the introduction of a NO_2_ moiety appeared related to the IN inhibitory activity, as compound **25** is the most active and selective of the five member ring series.

Interestingly, and conversely to what was previously observed for RNase H [30,31], the replacement of the dihydroxyphenyl core with other heteroaromatic nuclei had a greater impact on IN inhibitory activity.

As the indole moiety proved to be a key factor in some recently published IN allosteric inhibitors [43], we also examined compound **26**, whose activity against RT- RNase H has been reported [30]. Interestingly, it was most active against IN, although more selective for the RNase H function (Table 3), making it a good candidate for further optimization. Replacement of the indole moiety with pyrrole or 1-methyl pyrrole, as in compounds **27** and **28**, caused a loss of the IN activity and also affected RNAse H inhibition.

Finally, our attempt to investigate the mechanism of IN inhibition revealed the inability of the tested compounds to chelate Mg^2+^ ions. In fact, no Mg^2+^ effect was observed, as the absorbance peaks of our compounds did not show a significant shift with respect to control compound RDS1643, even at a high concentration of Mg^2+^ ions, suggesting a possible allosteric mode of action.

## 4. Materials and Methods

### 4.1. Chemistry

Unless otherwise specified, all reagents and solvents were obtained from commercial suppliers and used without further purification.

Progression of the reaction was monitored by thin-layer chromatography [Merck silica gel 60 pre-coated plates with fluorescent indicator F254 (0.25 mm)]. ^1^H and ^13^C NMR spectra were recorded on a Bruker Avance III™ HD 600 MHz spectrometer at 600 and 151 MHz, respectively. HPLC/APCI-MS analysis were recorded using a Varian tandem mass spectrometer (Palo Alto, CA, USA) 1200 L triple quadrupole mass spectrometer with an APCI source. Data acquisition and processing were performed by a Varian MS workstation version 6.7 software. APCI ran in both positive and negative ion modes. The capillary potential was set at 75 V, the APCI torch at 450 °C and the shield at 600 V. Nitrogen at 48 mTorr was fixed at 400 °C. The range of 100−1000 amu, with scan time 0.75 amu, scan width 0.70 amu, and detector at 1450 V were set to obtain the full scan spectra. The atmospheric pressure ionization (API) housing was kept at 50 °C for APCI. The compounds were solubilized in a mixture of acetonitrile 90% (*v*/*v*) (A) and doubly distilled water 10% (*v*/*v*) (B), and then infused in the source at a rate of 0.05 mL/min.

#### 4.1.1. General Procedure for the Synthesis of Thienopyrimidinones

***General procedure for synthesis of compounds* 10–12*,* 19–22*,* 24*,* 25*,* 27 *and* 28.**

Firstly, the 2-amino-4,5-disubstituted-thiophene-3-carboxamide intermediates were prepared as follows. A mixture of the appropriate ketone (20 mmol), cyanoacetamide (20 mmol), diethylamine (22 mmol, sulfur (20 mmol) and dry ethanol (20 mL) was stirred overnight at room temperature. After completion, the reaction was quenched with 140 mL of water and extracted five times with a mixture of ethyl acetate/ethanol (3/1). The organic layer, previously dried over Na_2_SO_4_, was filtered and the solvent was evaporated under vacuum to give a brown oil or solid. The crude product was then crystallized from ethanol, giving the corresponding intermediate. Then, the 2-amino-4,5-disubstituted-thiophene-3-carboxamide (1.8 mmol), molecular iodine (2.35 mmol), the proper aromatic aldehyde (2.2 mmol) and acetonitrile as solvent (25 mL) were stirred at room temperature for the period indicated (TLC) (1–5 h). After reaction, the mixture was quenched with 45 mL of 5% Na_2_S_2_O_3_ solution, and the resulting precipitate was collected by filtration under vacuum. The crude product was washed with petroleum ether/ethyl acetate (1:1) and recrystallized from ethanol to give the final products **10**–**12**, **19**–**22**, **24**, **25**, **27** and **28**. The purity of the compounds was assessed to be ≥95% by HPLC analysis (Agilent HP-1100, Santa Clara, CA, USA).

#### 4.1.2. Characterization Data

*2-(2,3-dihydroxyphenyl)-5,6-dimethylthieno[2,3-d]pyrimidin-4(3H)-one* (**10**). Yield (%) = 87. ^1^H NMR (600 MHz, DMSO) δ 12.14 (bs, 1H), 7.60 (d, *J* = 8.1 Hz, 1H), 6.98 (dd, *J* = 13.3, 7.3 Hz, 1H), 6.78 (t, *J* = 7.9 Hz, 1H), 2.41 (s, 3H), 2.37 (s, 3H) ppm. ^13^C NMR (151 MHz, DMSO) δ 158.51, 147.35, 146.72, 130.02, 129.32, 121.97, 119.51, 118.85, 118.83, 115.90, 13.29, 13.09 ppm. HRMS calculated for C_14_H_12_N_2_O_3_S 288.0569 found 288.0570.
*2-(2-hydroxyphenyl)-5,6-dimethylthieno[2,3-d]pyrimidin-4(3H)-one* (**11**). Yield (%) = 90. ^1^H NMR (600 MHz, DMSO) δ 12.14 (bs, 1H), 8.11 (d, *J* = 7.7 Hz, 1H), 7.42 (t, *J* = 7.8 Hz, 1H), 7.01 (d, *J* = 8.1 Hz, 1H), 6.98 (d, *J* = 7.6 Hz, 1H), 2.42 (s,3H), 2.39 (s, 3H) ppm. ^13^C NMR (151 MHz, DMSO) δ 160.84, 158.09, 153.26, 140.98, 133.63, 129.21, 119.95, 117.90, 111.57, 13.31, 13.12 ppm. HRMS calculated for C_14_H_12_N_2_O_2_S 272.0620 found 272.0621.
*2-(2,4-dihydroxyphenyl)-5,6-dimethylthieno[2,3-d]pyrimidin-4(3H)-one* (**12**). Yield (%) = 88. ^1^H NMR (600 MHz, DMSO) δ 12.15 (s, 1H), 8.48 (s, 1H), 8.04 (d, *J* = 8.5 Hz, 1H), 7.47 (s, *J* = 8.5 Hz, 1H), 2.40 (s, 3H), 2.36 (s, 3H) ppm. ^13^C NMR (151 MHz, DMSO) δ 62.96, 162.61, 157.42, 147.02, 132.93, 132.15, 130.42, 127.63, 112.70, 103.69, 102.90, 13.30, 13.08. HRMS calculated for C_14_H_12_N_2_O_3_S 288.0620 found 288.0619.
*2-(3,4-dihydroxyphenyl)-5-methyl-5,6,7,8-tetrahydrobenzo[4,5]thieno[2,3-d]pyrimidin-4(3H)-one* (**19**). Yield (%) = 82. ^1^H NMR (600 MHz, DMSO) δ 12.15 (bs, 1H), 9.63 (bs, 1H), 9.22 (bs, 1H), 7.58 (d, *J* = 2.2 Hz, 1H), 7.50 (dd, *J* = 8.7, 2.2 Hz, 1H), 6.81 (d, *J* = 8.7 Hz, 1H), 2.84–2.59 (m, 2H), 1.82 (s, 4H), 1.66 (d, *J* = 11.8 Hz, 1H), 1.25 (d, *J* = 6.9 Hz, 3H) ppm. ^13^C NMR (151 MHz, DMSO) δ 163.59, 160.32, 145.94, 145.83, 143.77, 137.71, 134.62, 134.12, 122.54, 121.29, 120.86, 115.44, 31.95, 30.36, 26.57, 25.80, 22.25 ppm. HRMS calculated for C_17_H_16_N_2_O_3_S 328.0882 found 328.0880.
*2-(3,4-dihydroxyphenyl)-5-isobutylthieno[2,3-d]pyrimidin-4(3H)-one* (**20**). Yield (%) = 80. ^1^H NMR (600 MHz, DMSO) δ 12.33 (bs, 1H), 9.72 (bs, 1H), 9.29 (bs, 1H), 7.55 (d, *J* = 2.4 Hz, 1H), 7.50 (d, *J* = 8.4 Hz, 1H), 6.85–6.79 (m, 1H), 3.00 (d, *J* = 7.1 Hz, 2H), 2.09–2.01 (m, 1H), 0.93 (d, *J* = 6.5 Hz, 6H) ppm. ^13^C NMR (151 MHz, DMSO) δ 168.66, 159.34, 155.61, 150.41, 149.41, 143.77, 137.71, 134.62, 123.41, 119.99, 115.95, 115.84, 35.40, 27.66, 23.90 ppm. HRMS calculated for C_16_H_16_N_2_O_3_S 316.0882 found 316.0881.
*2-(furan-2-yl)-5,6-dimethylthieno[2,3-d]pyrimidin-4(3H)-one* (**21**). Yield (%) = 92. ^1^H NMR (600 MHz, DMSO) δ 12.46 (s, 1H), 7.97 (d, *J* = 3.5 Hz, 1H), 7.58 (d, *J* = 3.5 Hz, 1H), 6.76–6.71 (m, 1H), 2.41 (s, 3H), 2.37 (s, 3H) ppm. ^13^C NMR (151 MHz, DMSO) δ162.53, 158.94, 156.49, 149.47, 144.38, 135.38, 129.37, 121.80, 116.11, 113.10, 109.52, 103.74, 13.32, 13.10 ppm. HRMS calculated for C_12_H_10_N_2_O_2_S 260.0463 found 260.0466.
*5,6-dimethyl-2-(5-methylfuran-2-yl)thieno[2,3-d]pyrimidin-4(3H)-one* (**22**). Yield (%) = 91. ^1^H NMR (600 MHz, DMSO) δ 12.33, 7.50, 7.50, 6.36, 6.36, 3.41, 2.52, 2.51, 2.51, 2.51, 2.50, 2.40, 2.39, 2.36 ppm. ^13^C NMR (151 MHz, DMSO) δ 162.53, 158.94, 156.49, 144.38, 144.14, 129.58, 129.37, 121.80, 116.11, 109.52, 13.99, 13.32, 13.10 ppm. HRMS calculated for C_13_H_12_N_2_O_2_S 260.0619 found 260.0621.
*5,6-dimethyl-2-(thiophen-2-yl)thieno[2,3-d]pyrimidin-4(3H)-one* (**24**). Yield (%) = 86. ^1^H NMR (600 MHz, DMSO) δ 12.61 (bs, 1H), 8.21 (d, *J* = 3.8 Hz, 1H), 7.85 (d, *J* = 5.1 Hz, 1H), 7.22 (ddd, *J* = 5.1, 3.8, 1.5 Hz, 1H), 2.41 (s, 3H), 2.37 (s, 3H) ppm. ^13^C NMR (151 MHz, DMSO) δ 155.26, 148.04, 147.97, 132.09, 132.03, 130.53, 130.18, 125.02, 122.61, 117.66,13.38, 13.27 ppm. HRMS calculated for C_17_H_16_N_2_OS_3_ 360.0425 found 360.0428.
*5,6-dimethyl-2-(5-nitrothiophen-2-yl)thieno[2,3-d]pyrimidin-4(3H)-one* (**25**). Yield (%) = 93. ^1^H NMR (600 MHz, DMSO) δ 7.84 (d, *J* = 4.0 Hz, 1H), 7.80 (d, *J* = 4.1 Hz, 1H), 2.41 (s, 3H), 2.39 (s, 3H) ppm. ^13^C NMR (151 MHz, DMSO) δ^13^C NMR (151 MHz, DMSO) δ 169.86, 163.66, 150.00, 143.74, 133.73, 131.06, 111.07, 103.24, 13.30, 13.10 ppm. HRMS calculated for C_12_H_9_N_3_O_3_S_2_ 307.0085 found 307.0088.
*5,6-dimethyl-2-(1H-pyrrol-2-yl)thieno[2,3-d]pyrimidin-4(3H)-one* (**27**). Yield (%) = 84. ^1^H NMR (600 MHz, DMSO) δ 12.13 (bs, 1H), 11.78 (bs, 1H), 7.28 (d, *J* = 3.8 Hz, 1H), 7.00 (d, *J* = 3.4 Hz, 1H), 6.20 (d, *J* = 3.4 Hz, 1H), 2.47–2.29 (m, 6H) ppm. ^13^C NMR (151 MHz, DMSO) δ 162.54, 158.95, 147.97, 134.24, 133.80, 129.26, 129.13, 125.28, 125.02, 13.11, 12.14 ppm. HRMS calculated for C_17_H_17_N_3_OS_2_ 343.0813 found 343.0816.
*5,6-dimethyl-2-(1-methyl-1H-pyrrol-2-yl)thieno[2,3-d]pyrimidin-4(3H)-one* (**28**). Yield (%) = 81. ^1^H NMR (600 MHz, DMSO) δ 12.40 (bs, 1H), 7.16 (d, *J* = 13.2 Hz, 1H), 7.06 (d, *J* = 13.2 Hz, 1H), 6.13 (d, *J* = 3.2 Hz, 1H), 3.62 (s, 3H), 2.50 (s, 3H), 2.36 (s, 3H) ppm. ^13^C NMR (151 MHz, DMSO) δ 166.82, 152.52, 145.94, 145.83, 134.62, 134.12, 120.54, 119.99, 118.83, 115.84, 41.13, 12.07 ppm. HRMS calculated for C_18_H_19_N_3_OS_2_ 357.0970 found 357.0973.

### 4.2. Biology

#### 4.2.1. Expression and Purification of HIV-1 Integrase and LEDGF/p75 Proteins

Recombinant His and FLAG-IN proteins and His and FLAG-LEDGF proteins were expressed in *Escherichia coli* strain BL21 (DE3) and purified, as previously described [44].

#### 4.2.2. HTRF LEDGF-Dependent Assays

IN activity in the presence of recombinant LEDGF/p75 protein were determined as described before [45]. IN was pre-incubated in reaction buffer containing 20 mM HEPES pH 7.5, 1 mM DTT, 1% glycerol, 20 mM MgCl_2_, 0.05% Brij-35 and 0.1 mg/mL BSA with increasing concentration of compounds for 1 h at room temperature. After the addition to this mixture of 9 nM DNA donor substrate, 50 nM DNA acceptor substrate and 50 nM LEDGF/p75 protein (when required), the reaction was incubated at 37 °C for 90 min. The addition of 4 nM of Europium–Streptavidin to the reaction mixture provided the HTRF signal, which was recorded using a Perkin Elmer (Waltham, MA, USA) Victor 3 plate reader with 314 nm for excitation wavelength and 668 and 620 nm for the wavelength of the acceptor and the donor substrates emission, respectively.

#### 4.2.3. MgCl_2_ Coordination Assay

The ability of the compounds to coordinate Mg^2+^ were determined by means of previously reported protocols [31]. The UV–Vis spectrum from 250 to 600 nm was recorded before and after the addition of 6 mM MgCl_2_ and plotted using Prism9.

#### 4.2.4. RNase H inibitory Assay

The previously reported procedure was used for preparation and purification of wild type p66/p51 HIV-1 RT. Enzyme was stored in a 50% glycerol-containing buffer at −20 °C [32,46]. An 18-nt 3′-fluorescein-labeled RNA annealed to a complementary 18-nt 5′-dabsyl-labeled DNA was used to determine IC_50_ values, following a previously described procedure [47]. In brief, 1 μL of each inhibitor dissolved in DMSO was added to a 96- well plate, followed by 10 μL of the proper RT (15−80 ng/mL) in reaction buffer. The addition of 10 μL of RNA/DNA hybrid (2.5 μM) initiated the hydrolysis. Then, 50 mM Tris-HCl, pH 8.0, 60 mM KCl, 10 mM MgCl_2_, 1% DMSO, 150−800 ng of RT, 250 nM substrate, and increasing concentrations of inhibitor were chosen as final assay conditions. As negative control, wells containing only DMSO were used. Subsequently, the plates were incubated in a Spectramax Gemini EM fluorescence spectrometer for 10 min at 37 °C, measuring the fluorescence (λex = 485 nm; λem = 520 nm) at 1 min intervals in order to determine the linear initial rates in the presence (vi) and absence (vo) of inhibitor. Percent inhibition was assessed as 100(vo − vi)/vo and plotted against log[I]. Prism5 (GraphPad Software 5.0) was used to calculate the IC_50_ values. All assays were carried out in triplicate.

## 5. Conclusions

Since the dihydroxyphenyl unit has been evidenced in various allosteric IN inhibitors, we investigated here the activity of novel thienopyrimidinones bearing a dihydrohyphenyl core at C-2. Our findings showed a strict correlation between the OH position and their IN inhibitory activity, and coordination assays evidenced no Mg^2+^ effect, suggesting an allosteric mode of action.

In conclusion, the thienopyrimidinone skeleton is confirmed as a privileged framework in HIV drug discovery. Furthermore, as catechol may be implicated in cytotoxicity, we also explored its substitution with other heteroaromatic scaffolds. In particular, our findings indicated that variation at C-2 position may be a key factor for compound selectivity. In this regard, compounds **21**–**23** and **25**–**26** with an improved activity were revealed to be a good hit to lead candidates. In particular, compound **26** showed very good activity on both RNase H and IN and is proposed as a dual-inhibitor, while 5,6-dimethyl-2-(5-nitrothiophen-2-yl)thieno[2,3-d]pyrimidin-4(3H)-one **25**, which was completely inactive on RNase H but with a good IN IC_50_, was the most selective against IN.

Substitutions at C-5 and C-6 also proved to be vital. In this regard, the crucial introduction of a branched cyclohexyl moiety instead of a cycloheptane ring offered the 2-(3,4-dihydroxyphenyl)-5-methyl-5,6,7,8-tetrahydrobenzo[4,5]thieno[2,3-d]pyrimidin-4(3H)-one **20** as a hit compound for further exploitation. Encouraged by the results, novel derivatives are now under consideration, also using an in silico approach, to gain more insight into the allosteric binding site (manuscript in preparation).

## Figures and Tables

**Table 1:** Effect of thienopyrimidinones **8**, **10**–**14** on the HIV-1 IN LEDGF/p75-dependent activity.

	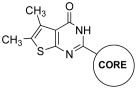	
Compound	CORE	IC_50_ (μM)
**8** [30]	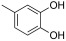	24.4 ± 2.60
**10**	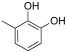	12.6 ± 1.29
**11**	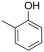	21.96 ± 2.8
**12**	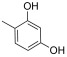	22.26 ± 1.57
**13**	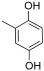	85 ± 6.00
**14**	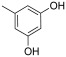	3.7 ± 0.73
**RAL**		0.055 ± 0.002

**Table 2:** Effect of thienopyrimidinones **9**, **15**–**18** on the HIV-1 IN LEDGF/p75-dependent activity.

	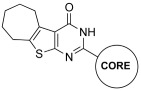	
Compound	CORE	IC_50_ (μM)
**9** [30]	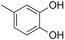	18.3 ± 2.3
**15**	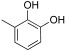	24 ± 1
**16**	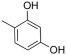	10 ± 1.00
**17**	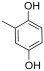	5.65 ± 0.15
**18**	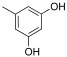	3.37 ± 0.48
**RAL**		0.055 ± 0.002

**Table 3:** Effect of the thiophene ring substitution on the HIV-1 IN LEDGF/p75-dependent activity.

Compound	STRUCTURE	IC_50_ (μM)
**19**	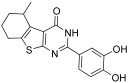	5.9 ± 0.5
**20**	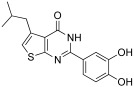	8.5 ± 2.5
**RAL**		0.055 ± 0.002

**Table 4:** Effect of thienopyrimidinones **21**–**28** on the HIV-1 IN LEDGF/p75-dependent activity and RT-RNase H.

	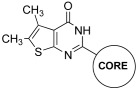		
Compound	CORE	IC_50_ (μM)	
		IN	RNase-H
**21**	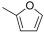	5.75 ± 0.85	2.5 ± 0.1
**22**	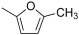	8.75 ± 0.35	14.4 ± 0.2
**23**	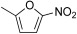	4.6 ± 0.82	16.9 ± 1.7 [30]
**24**	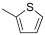	>50	8.2 ± 1.3
**25**	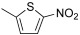	3.2 ± 0.80	>50
**26**	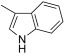	2.85 ± 0.85	0.48 ± 0.6 [30]
**27**	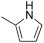	>50	35.3 ± 1.5
**28**	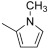	>50	14.8 ± 0.6
**RAL**		0.055 ± 0.002	–
**GZ 510** [30]		–	0.26 ± 0.01

## Data Availability

The data presented in this study are available within the article and in the Supplementary Material.

## Supplementary Materials

The following supporting information can be downloaded at: https://www.mdpi.com/article/10.3390/molecules28186700/s1, ^1^H and ^13^C NMR spectra: pp. 2–23. Mg^2+^ spectra: pp. 24–32.

## References

[1] Data on the Size of the HIV Epidemic. https://www.who.int/data/gho/data/themes/hiv-aids/data-on-the-size-of-the-hiv-aids-epidemic.

[2] Anthony N.J. (2004). HIV-1 integrase: A target for new AIDS chemotherapeutics. Curr. Top. Med. Chem..

[3] Dyda F., Hickman A.B., Jenkins T.M., Engelman A., Craigie R., Davies D.R. (1994). Crystal structure of the catalytic domain of HIV-1 integrase: Similarity to other polynucleotidyl transferases. Science.

[4] Esposito D., Craigie R. (1999). HIV integrase structure and function. Adv. Virus Res..

[5] Krishnan L., Engelman A. (2012). Retroviral integrase proteins and HIV-1 DNA integration. J. Biol. Chem..

[6] Kassahun K., McIntosh I., Cui D., Hreniuk D., Merschman S., Lasseter K., Azrolan N., Iwamoto M., Wagner J.A., Wenning L.A. (2007). Metabolism and disposition in humans of Raltegravir (MK-0518), an anti-AIDS drug targeting the human immunodeficiency virus 1 integrase enzyme. Drug Metab. Dispos..

[7] Summa V., Petrocchi A., Bonelli F., Crescenzi B., Donghi M., Ferrara M., Fiore F., Gardelli C., Gonzalez Paz O., Hazuda D.J. (2008). Discovery of Raltegravir, a potent, selective orally bioavailable HIV-integrase inhibitor for the treatment of HIV-AIDS infection. J. Med. Chem..

[8] Marchand C. (2012). The Elvitegravir Quad pill: The first once-daily dual-target anti-HIV tablet. Expert Opin. Investig. Drugs.

[9] Mercadel C.J., Skelley J.W., Kyle J.A., Elmore L.K. (2014). Dolutegravir: An integrase strand transfer inhibitor for the treatment of human immunodeficiency virus 1 in adults. J. Pharm. Technol..

[10] Taki E., Soleimani F., Asadi A., Ghahramanpour H., Namvar A., Heidary M. (2022). Cabotegravir/Rilpivirine: The last FDA-approved drug to treat HIV. Expert Rev. Anti Infect. Ther..

[11] Spagnuolo V., Castagna A., Lazzarin A. (2018). Bictegravir. Curr. Opin. HIV AIDS.

[12] Cherepanov P., Maertens G., Proost P., Devreese B., Van Beeumen J., Engelborghs Y., De Clercq E., Debyse Z. (2003). HIV-1 integrase forms stable tetramers and associates with LEDGF/p75 protein in human cells. J. Biol. Chem..

[13] Busschots K., Vercammen J., Emiliani S., Benarous R., Engelborghs Y., Christ F., Debyser Z. (2005). The interaction of LEDGF/p75 with integrase is lentivirus-specific and promotes DNA binding. J. Biol. Chem..

[14] Cherepanov P., Ambrosio A.L., Rahman S., Ellenberger T., Engelman A. (2005). Structural basis for the recognition between HIV-1 integrase and transcriptional coactivator p75. Proc. Natl. Acad. Sci. USA.

[15] Ciuffi A., Llano M., Poeschla E., Hoffmann C., Leipzig J., Shinn P., Ecker J.R., Bushman F. (2005). A role for LEDGF/p75 in targeting HIV DNA integration. Nat. Med..

[16] Llano M., Saenz D.T., Meehan A., Wongthida P., Peretz M., Walker W.H., Teo W., Poeschla E.M. (2006). An essential role for LEDGF/p75 in HIV integration. Science.

[17] Shun M.C., Raghavendra N.K., Vandegraaff N., Daigle J.E., Hughes S., Kellam P., Cherepanov P., Engelman A. (2007). LEDGF/p75 functions downstream from preintegration complex formation to effect gene-specific HIV-1 integration. Genes Dev..

[18] Ferris A.L., Wu X., Hughes C.M., Stewart C., Smith S.J., Milne T.A., Wang G.G., Shun M.C., Allis C.D., Engelman A. (2010). Lens epithelium-derived growth factor fusion proteins redirect HIV-1 DNA integration. Proc. Natl. Acad. Sci. USA.

[19] Kessl J.J., Jena N., Koh Y., Taskent-Sezgin H., Slaughter A., Feng L., de Silva S., Wu L., Le Grice S.F.J., Engelman A. (2012). Multimode, cooperative mechanism of action of allosteric HIV-1 integrase inhibitors. J. Biol. Chem..

[20] Jurado K.A., Engelman A. (2013). Multimodal mechanism of action of allosteric HIV-1 integrase inhibitors. Expert Rev. Mol. Med..

[21] Jurado K.A., Wang H., Slaughter A., Feng L., Kessl J.J., Koh Y., Wang W., Ballandras-Colas A., Patel P.A., Fuchs J.R. (2013). Allosteric integrase inhibitor potency is determined through the inhibition of HIV-1 particle maturation. Proc. Natl. Acad. Sci. USA.

[22] Shkriabai N., Dharmarajan V., Slaughter A., Kessl J.J., Larue R.C., Feng L., Fuchs J.R., Griffin P.R., Kvaratskhelia M. (2014). A critical role of the C-terminal segment for allosteric inhibitor-induced aberrant multimerization of HIV-1 integrase. J. Biol. Chem..

[23] Koneru P.C., Francis A.C., Deng N., Rebensburg S.V., Hoyte A.C., Lindenberger J., Adu-Ampratwum D., Larue R.C., Wempe M.F., Engelman A.N. (2019). HIV-1 integrase tetramers are the antiviral target of pyridine-based allosteric integrase inhibitors. eLife.

[24] Feng L., Sharma A., Slaughter A., Jena N., Koh Y., Shkriabai N., Larue R.C., Patel P.A., Mitsuya H., Kessl J.J. (2013). The A128T Resistance Mutation Reveals Aberrant Protein Multimerization as the Primary Mechanism of Action of Allosteric HIV-1 Integrase Inhibitors. J. Biol. Chem..

[25] Adu-Ampratwum D., Pan Y., Koneru P.C., Antwi J., Hoyte A.C., Kessl J., Griffin P.R., Kvaratskhelia M., Fuchs J.R., Larue R.C. (2022). Identification and Optimization of a Novel HIV-1 Integrase Inhibitor. ACS Omega.

[26] Andrae-Marobela K., Ghislain F.W., Okatch H., Majinda R.R. (2013). Polyphenols: A diverse class of multi-target anti-HIV-1 agents. Curr. Drug Metab..

[27] Di Petrillo A., Orrù G., Fais A., Fantini M.C. (2022). Quercetin and its derivates as antiviral potentials: A comprehensive review. Phytother. Res..

[28] Ngoutane Mfopa A., Corona A., Eloh K., Tramontano E., Frau A., Boyom F.F., Caboni P., Tocco G. (2018). Uvaria angolensis as a promising source of inhibitors of HIV-1 RT-associated RNA-dependent DNA polymerase and RNase H functions. Nat. Prod. Res..

[29] Esposito F., Tintori C., Martini R., Christ F., Debyser Z., Ferrarese R., Cabiddu G., Corona A., Ceresola E.R., Calcaterra A. (2015). Kuwanon-L as a New Allosteric HIV-1 Integrase Inhibitor: Molecular Modeling and Biological Evaluation. Chembiochem.

[30] Masaoka T., Chung S., Caboni P., Rausch J.W., Wilson J.A., Taskent-Sezgin H., Beutler J.A., Tocco G., Le Grice S.F. (2013). Exploiting drug-resistant enzymes as tools to identify thienopyrimidinone inhibitors of human immunodeficiency virus reverse transcriptase-associated ribonuclease H. J. Med. Chem..

[31] Corona A., Masaoka T., Tocco G., Tramontano E., Le Grice S.F. (2013). Active site and allosteric inhibitors of the ribonuclease H activity of HIV reverse transcriptase. Future Med. Chem..

[32] Tocco G., Esposito F., Caboni P., Laus A., Beutler J., Wilson J., Corona A., Le Grice S.F.J., Tramontano E. (2020). Scaffold hopping and optimisation of 3′,4′-dihydroxyphenyl- containing thienopyrimidinones: Synthesis of quinazolinone derivatives as novel allosteric inhibitors of HIV-1 reverse transcriptase-associated ribonuclease H. J. Enzym. Inhib. Med. Chem..

[33] Abdelaziz O.A., El Husseiny W.M., Selim K.B., Eisa H.M. (2022). Synthesis, Antitumor Activity, and In Silico Drug Design of New Thieno[2,3-d]Pyrimidine-4-One Derivatives as Nonclassical Lipophilic Dihydrofolate Reductase Inhibitors. ACS Omega.

[34] Mustière R., Lagardère P., Hutter S., Deraeve C., Schwalen F., Amrane D., Masurier N., Azas N., Lisowski V., Verhaeghe P. (2022). Pd-catalyzed C-C and C-N cross-coupling reactions in 2-aminothieno[3,2-d]pyrimidin-4(3H)-one series for antiplasmodial pharmacomodulation. RSC Adv..

[35] Bosson-Vanga H., Primas N., Franetich J.F., Lavazec C., Gomez L., Ashraf K., Tefit M., Soulard V., Dereuddre-Bosquet N., Le Grand R. (2021). A New Thienopyrimidinone Chemotype Shows Multistage Activity against Plasmodium falciparum, Including Artemisinin-Resistant Parasites. Microbiol. Spectr..

[36] Magoulas G.E., Kalopetridou L., Ćirić A., Kritsi E., Kouka P., Zoumpoulakis P., Chondrogianni N., Soković M., Prousis K.C., Calogeropoulou T. (2021). Synthesis, biological evaluation and QSAR studies of new thieno[2,3-d]pyrimidin-4(3H)-one derivatives as antimicrobial and antifungal agents. Bioorg. Chem..

[37] Bekhit A.A., Farghaly A.M., Shafik R.M., Elsemary M.M.A., Bekhit A.E.A., Guemei A.A., El-Shoukrofy M.S., Ibrahim T.M. (2018). Synthesis, biological evaluation and molecular modeling of novel thienopyrimidinone and triazolothienopyrimidinone derivatives as dual anti-inflammatory antimicrobial agents. Bioorg. Chem..

[38] Pisal M.M., Nawale L.U., Patil M.D., Bhansali S.G., Gajbhiye J.M., Sarkar D., Chavan S., Borate H.B. (2017). Hybrids of thienopyrimidinones and thiouracils as anti-tubercular agents: SAR and docking studies. Eur. J. Med. Chem..

[39] Baell J.B., Holloway G.A. (2010). New substructure filters for removal of pan assay interference compounds (PAINS) from screening libraries and for their exclusion in bioassays. J. Med. Chem..

[40] Ingólfsson H.I., Thakur P., Herold K.F., Hobart E.A., Ramsey N.B., Periole X., de Jong D.H., Zwama M., Yilmaz D., Hall K. (2014). Phytochemicals perturb membranes and promiscuously alter protein function. ACS Chem. Biol..

[41] Jóźwik I.K., Passos D.O., Lyumkis D. (2020). Structural Biology of HIV Integrase Strand Transfer Inhibitors. Trends Pharmacol. Sci..

[42] Shea K.M., Li J.J., Gribble G.W. (2007). Furans and benzo[*b*]furans. Palladium in Heterocyclic Chemistry. A Guide for the Synthetic Chemist.

[43] Patel P.A., Kvaratskhelia N., Mansour Y., Antwi J., Feng L., Koneru P., Kobe M.J., Jena N., Shi G., Mohamed M.S. (2016). Indole-based allosteric inhibitors of HIV-1 integrase. Bioorg. Med. Chem. Lett..

[44] Esposito F., Ambrosio F.A., Meleddu R., Costa G., Rocca R., Maccioni E., Catalano R., Romeo I., Eleftheriou P., Karia D.C. (2019). Chromenone Derivatives as a Versatile Scaffold with Dual Mode of Inhibition of HIV-1 Reverse Transcriptase-Associated Ribonuclease H Function and Integrase Activity. Eur. J. Med. Chem..

[45] Sanna C., Rigano D., Corona A., Piano D., Formisano C., Farci D., Franzini G., Ballero M., Chianese G., Tramontano E. (2019). Dual HIV-1 Reverse Transcriptase and Integrase Inhibitors from Limonium morisianum Arrigoni, an Endemic Species of Sardinia (Italy). Nat. Prod. Res..

[46] Chung S., Miller J.T., Johnson B.C., Hughes S.H., Le Grice S.F. (2012). Mutagenesis of human immunodeficiency virus reverse transcriptase p51 subunit defines residues contributing to vinylogous urea inhibition of ribonuclease H activity. J. Biol. Chem..

[47] Chung S., Wendeler M., Rausch J.W., Beilhartz G., Gotte M., O’Keefe B.R., Bermingham A., Beutler J.A., Liu S., Zhuang X. (2010). Structure-activity analysis of vinylogous urea inhibitors of human immunodeficiency virus-encoded ribonuclease H. Antimicrob. Agents Chemother..

