# Supplementary material for: Dihydroxyphenyl- and Heteroaromatic-Based Thienopyrimidinones to Tackle HIV-1 LEDGF/p75-Dependent IN Activity
Source: Molecules. 2023 Sep 19;28(18):6700. doi: 10.3390/molecules28186700 (PMC10537185; doi:10.3390/molecules28186700)
Supplement: Supplementary file 1 [file molecules-28-06700-s001.zip › molecules-2514940-supplementary.pdf]

Dihydroxyphenyl- and heteroaromatic-based thienopyrimidinones to tackle HIV-1 LEDGF/p75-dependent IN activity.

Graziella Tocco[1,*], Serena Canton[1], Antonio Laus[1,2], Pierluigi Caboni[1], Stuart F.J. Le Grice[3], Enzo Tramontano[1] and Francesca Esposito[1]

1 Department of Life and Environmental Sciences, University of Cagliari, Cittadella Universitaria di Monserrato, Monserrato, Cagliari, Italy; toccog@unica.it (G.T); serena3canton@gmail.com (S.C.);caboni@unica.it (P.C.); tramon@unica.it (E.T.); francescaesposito@unica.it (F.E.).

2 Department of Life Sciences, University of Modena and Reggio Emilia, Modena, Italy; antolaus@unimore.it (current address)

3 Center for Cancer Research, National Cancer Institute, 21702-1201, Frederick, MD, USA; garysbruv@gmail.com

* Correspondence: toccog@unica.it;

Table of contents

- ^{1}H and ^{13}C NMR spectra of compounds **10-12, 19 -22, 24, 25, 27** and **28**…………pp.2-23
- Mg^{2+} spectra of compounds **10-23, 25** and **26**……………………… ……………..……pp.24-32

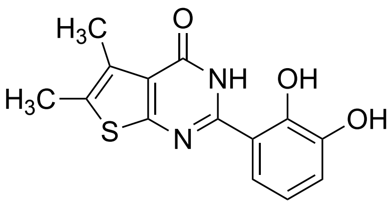

10

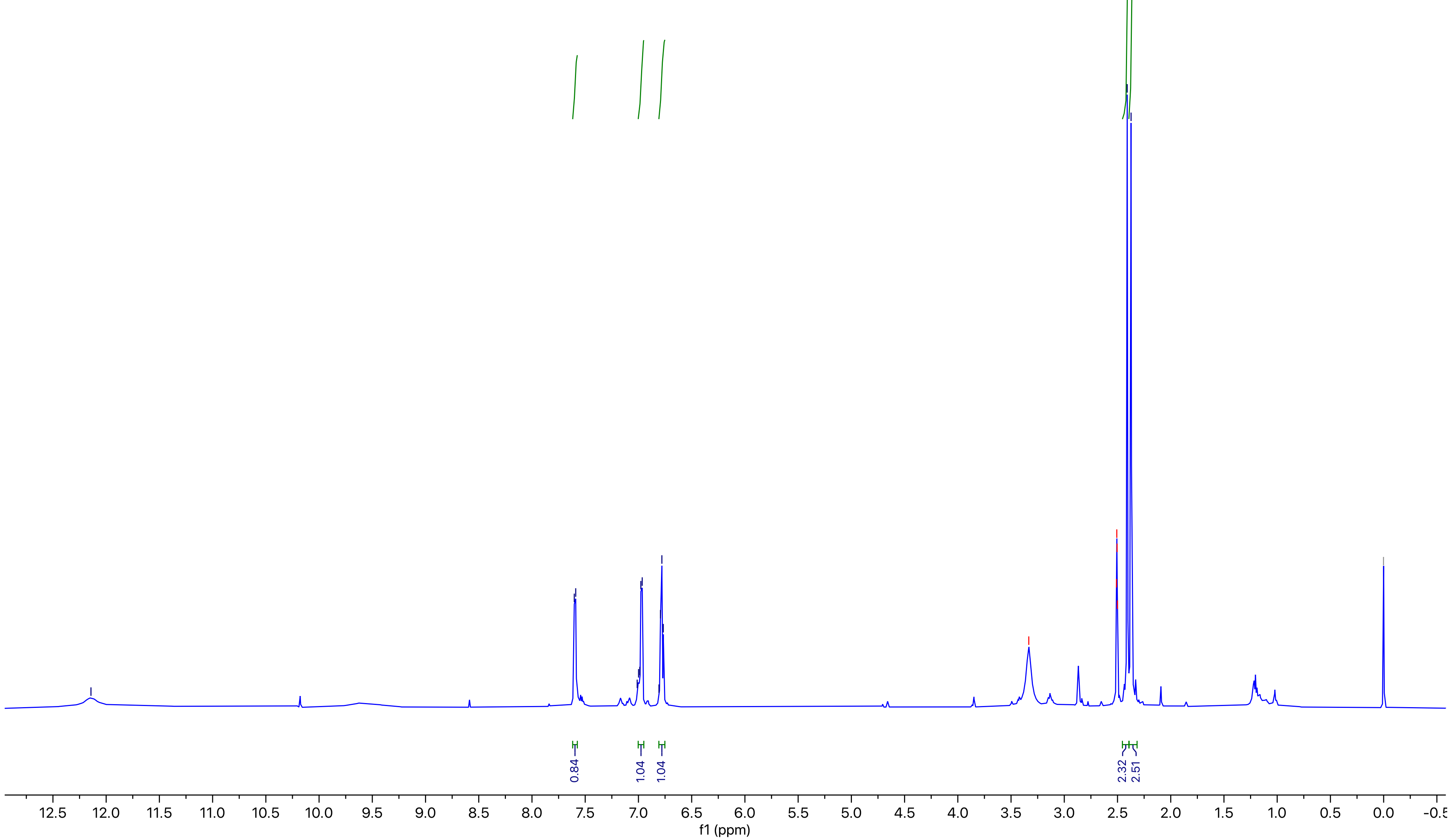
12.14
7.60
7.59
7.01
7.00
6.99
6.98
6.96
6.81
6.79
6.78
6.77
3.33 H2O
2.51 DMSO
2.51 DMSO
2.50 DMSO
2.50 DMSO
2.41
2.37
-0.00
0.84
1.04
1.04
2.32
2.51
12.5 12.0 11.5 11.0 10.5 10.0 9.5 9.0 8.5 8.0 7.5 7.0 6.5 6.0 5.5 5.0 4.5 4.0 3.5 3.0 2.5 2.0 1.5 1.0 0.5 0.0 -0.5
f1 (ppm)

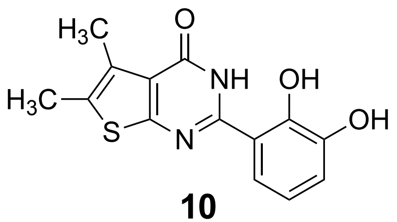

10

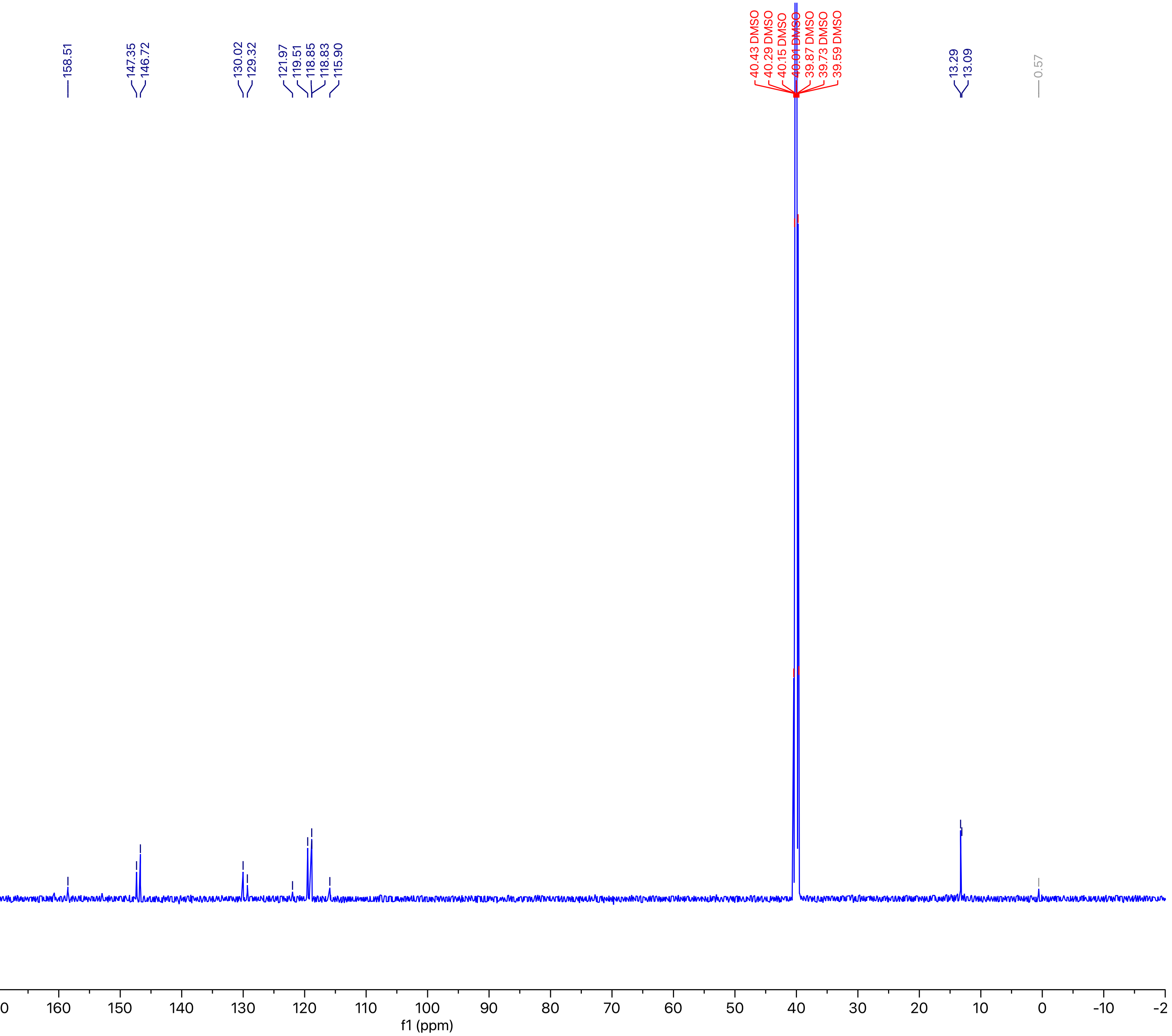
158.51
147.35
146.72
130.02
129.32
121.97
119.51
118.85
118.83
115.90
40.43 DMSO
40.29 DMSO
40.15 DMSO
40.01 DMSO
39.87 DMSO
39.73 DMSO
39.59 DMSO
13.29
13.09
0.57
f1 (ppm)

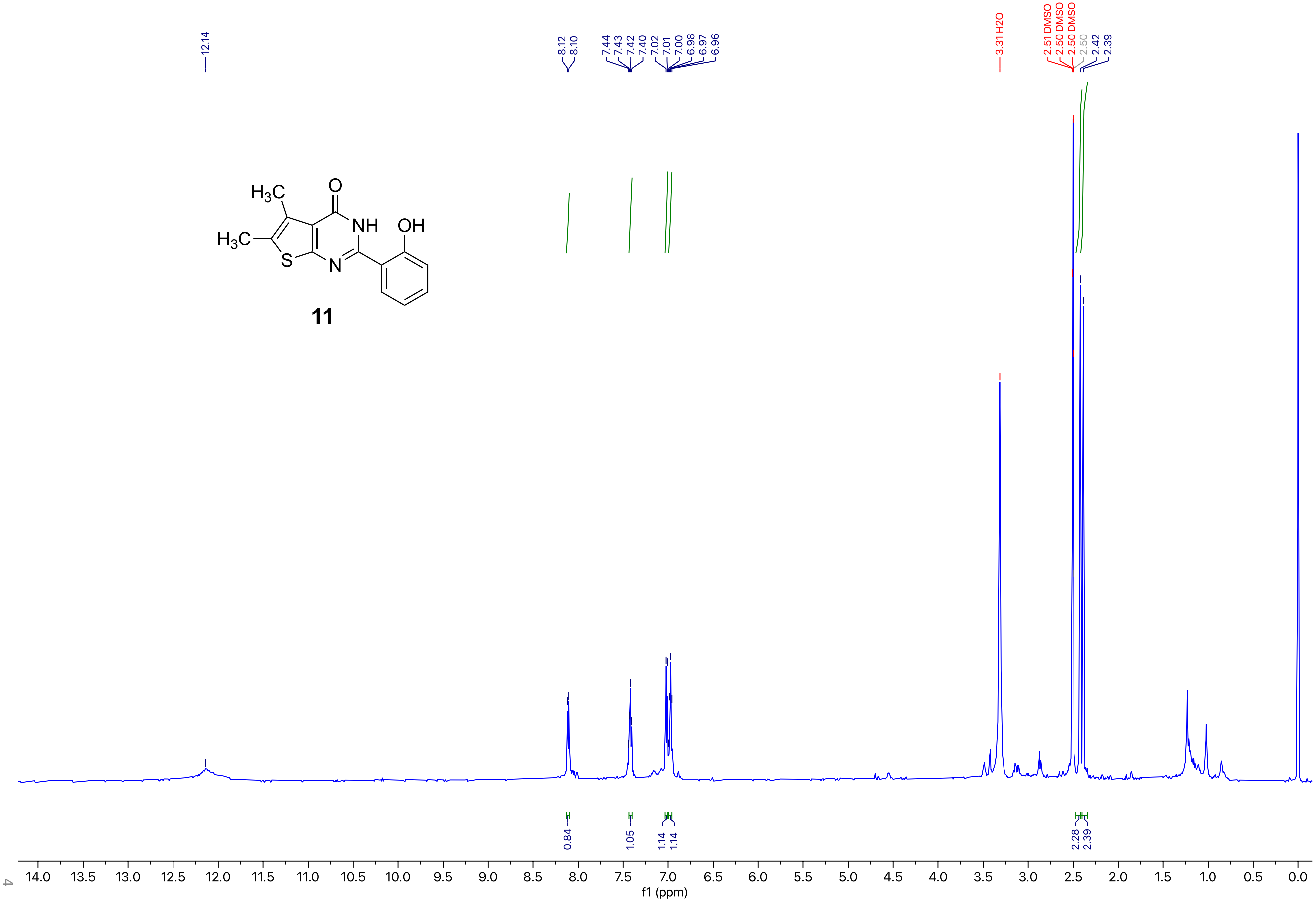
12.14
8.12
8.10
7.44
7.43
7.42
7.40
7.02
7.01
7.00
6.98
6.97
6.96
3.31 H2O
2.51 DMSO
2.50 DMSO
2.50 DMSO
2.50
2.42
2.39
H3C
H3C
O
NH
OH
S
N
11
0.84
1.05
1.14
1.14
2.28
2.39
14.0 13.5 13.0 12.5 12.0 11.5 11.0 10.5 10.0 9.5 9.0 8.5 8.0 7.5 7.0 6.5 6.0 5.5 5.0 4.5 4.0 3.5 3.0 2.5 2.0 1.5 1.0 0.5 0.0
f1 (ppm)

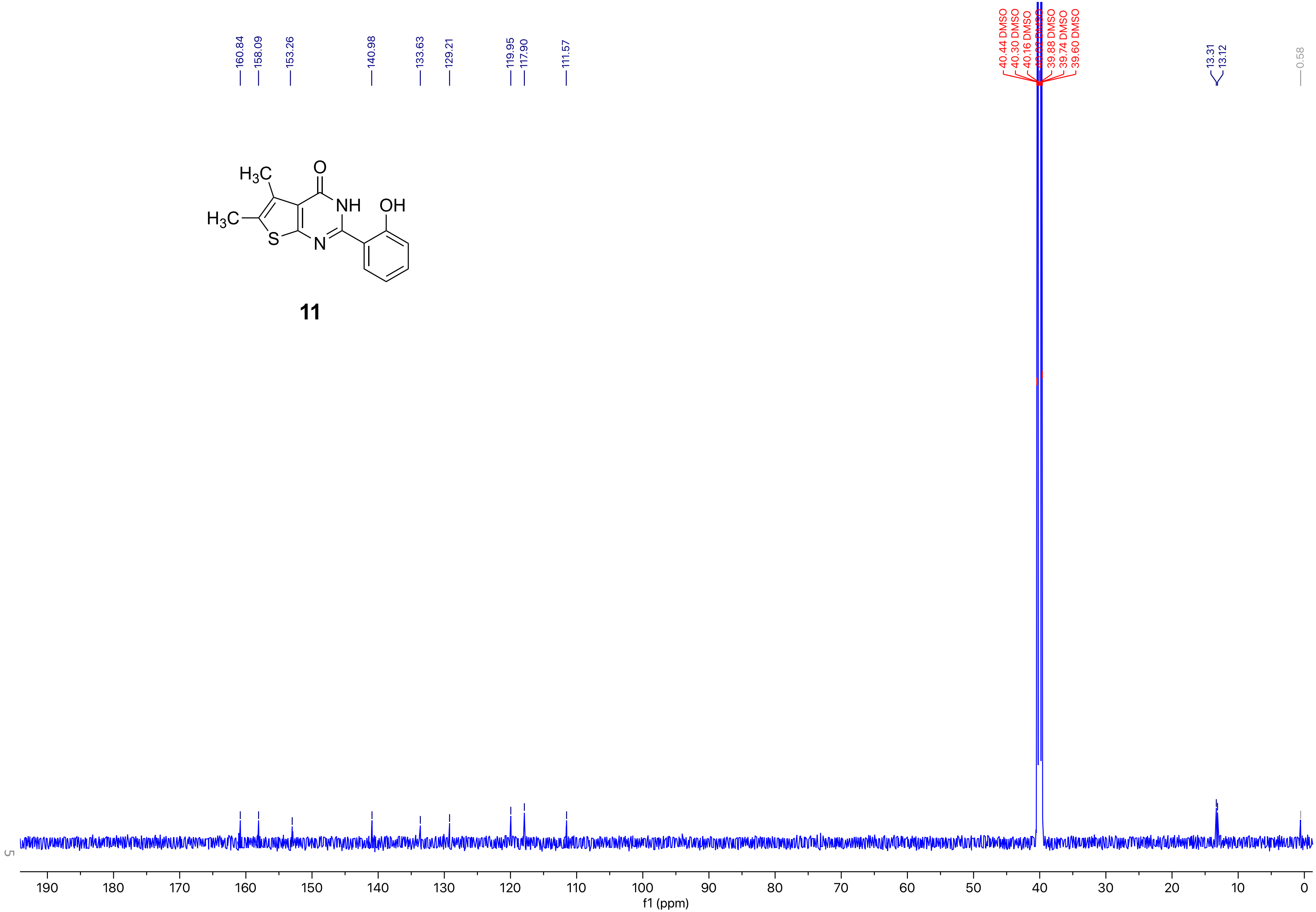
160.84
158.09
153.26
140.98
133.63
129.21
119.95
117.90
111.57
40.44 DMSO
40.30 DMSO
40.16 DMSO
39.88 DMSO
39.74 DMSO
39.60 DMSO
13.31
13.12
0.58
H3C
O
NH
OH
S
N
11
190 180 170 160 150 140 130 120 110 100 90 80 70 60 50 40 30 20 10 0
f1 (ppm)

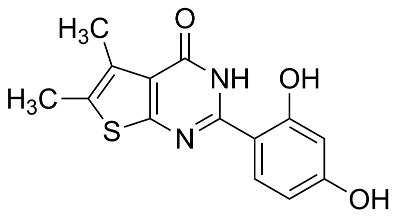
H3C
H3C
O
NH
OH
S
N
OH

12

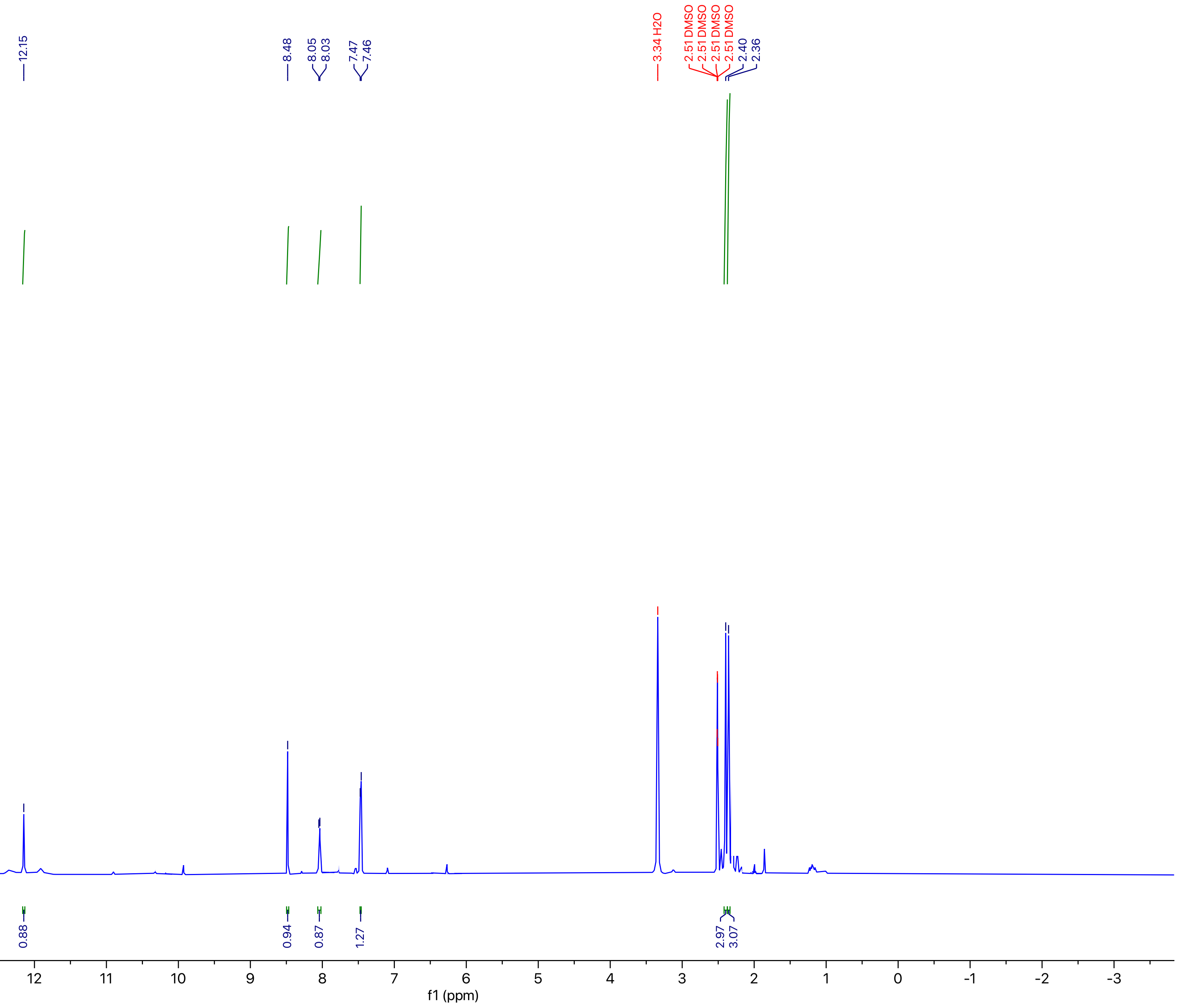
12.15
8.48
8.05
8.03
7.47
7.46
3.34 H2O
2.51 DMSO
2.51 DMSO
2.51 DMSO
2.51 DMSO
2.40
2.36
0.88
0.94
0.87
1.27
2.97
3.07
16
15
14
13
12
11
10
9
8
7
6
5
4
3
2
1
0
-1
-2
-3
f1 (ppm)

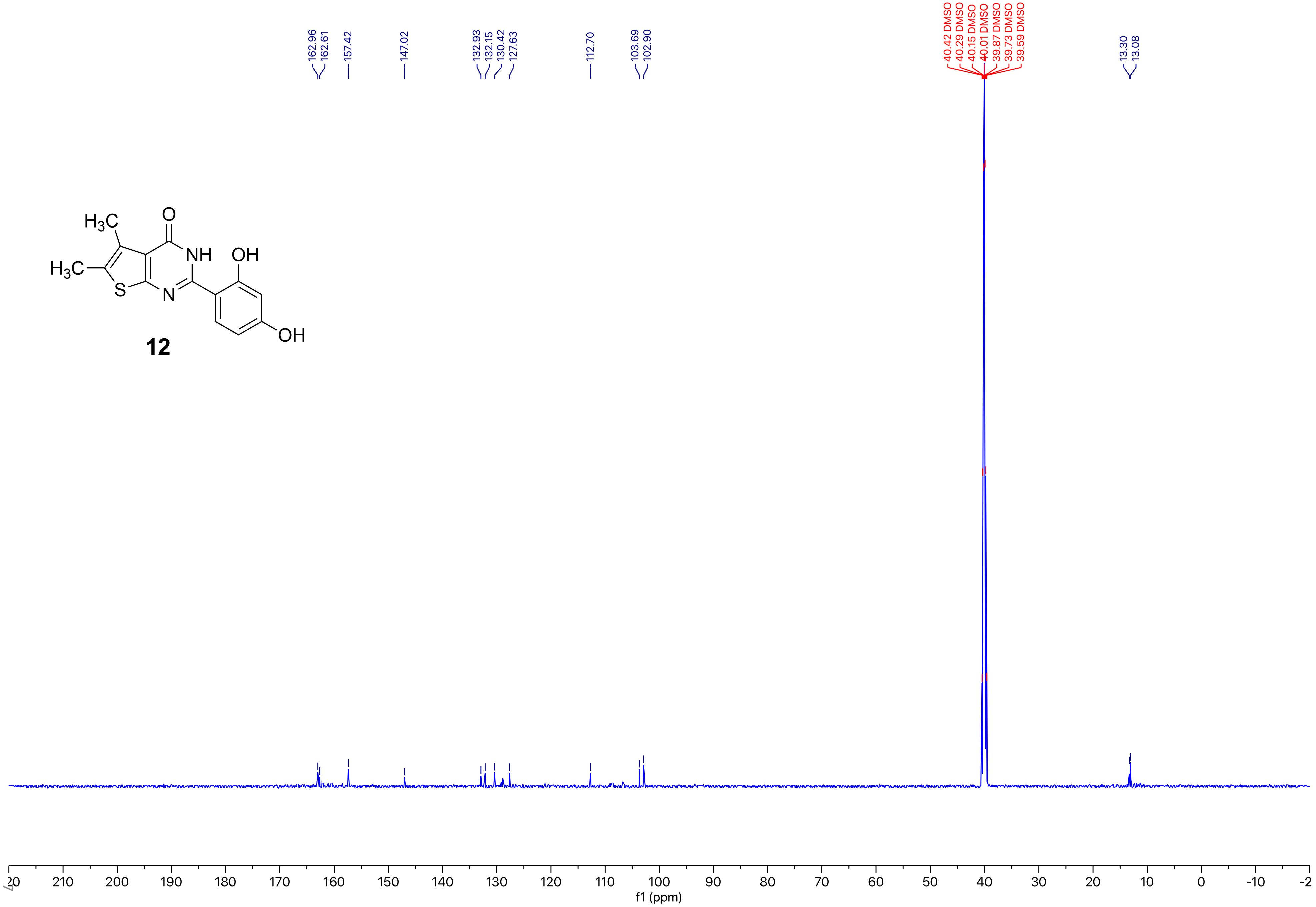
12
162.96
162.61
157.42
147.02
132.93
132.15
130.42
127.63
112.70
103.69
102.90
40.42 DMSO
40.29 DMSO
40.15 DMSO
40.01 DMSO
39.87 DMSO
39.73 DMSO
39.59 DMSO
13.30
13.08
f1 (ppm)

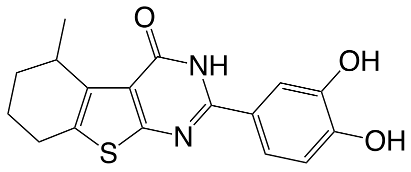

19

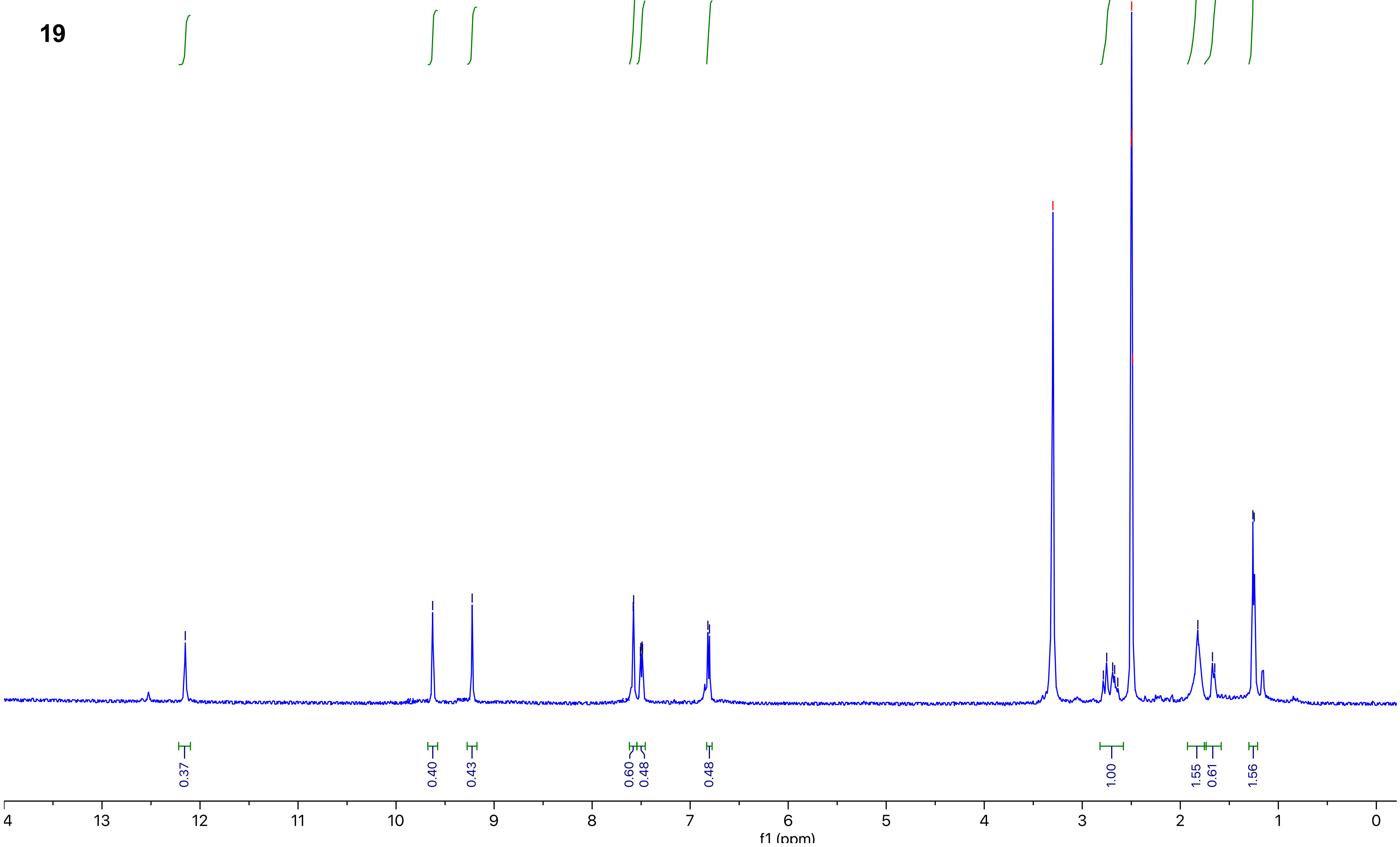
12.15
9.63
9.22
7.58
7.58
7.51
7.50
7.49
7.49
6.82
6.80
3.30 H2O
2.78
2.75
2.69
2.67
2.64
2.50 dmsc
2.50 dmsc
2.49 dmsc
2.49 dmsc
1.82
1.67
1.65
1.26
1.25
0.37
0.40
0.43
0.60
0.48
0.48
1.00
1.55
0.61
1.56
f1 (ppm)

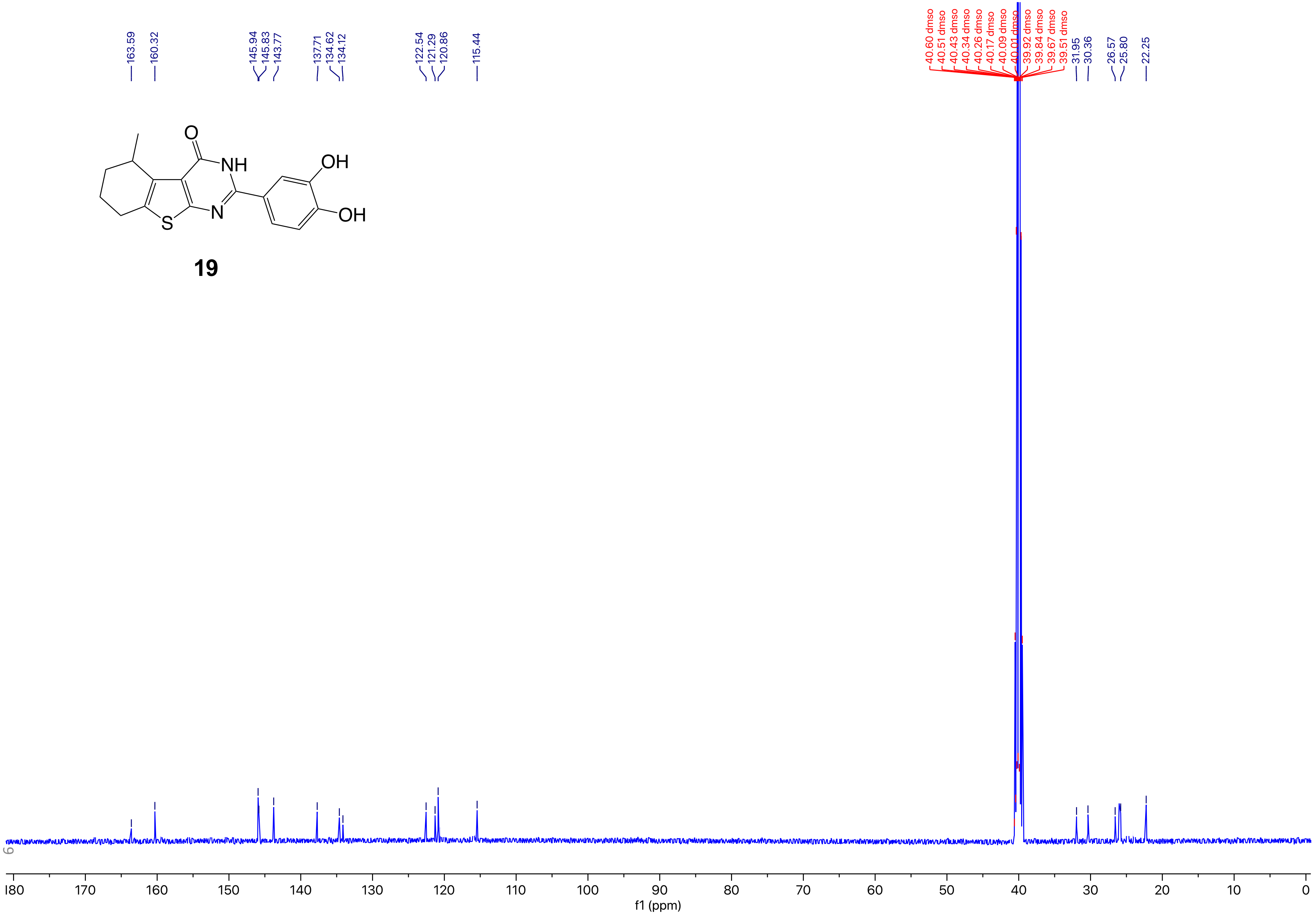

163.59
160.32
145.94
145.83
143.77
137.71
134.62
134.12
122.54
121.29
120.86
115.44
40.60 dmso
40.51 dmso
40.43 dmso
40.34 dmso
40.26 dmso
40.17 dmso
40.09 dmso
40.01 dmso
39.92 dmso
39.84 dmso
39.67 dmso
39.51 dmso
31.95
30.36
26.57
25.80
22.25
O
NH
OH
OH
N
S
19
180
170
160
150
140
130
120
110
100
90
80
70
60
50
40
30
20
10
0
f1 (ppm)

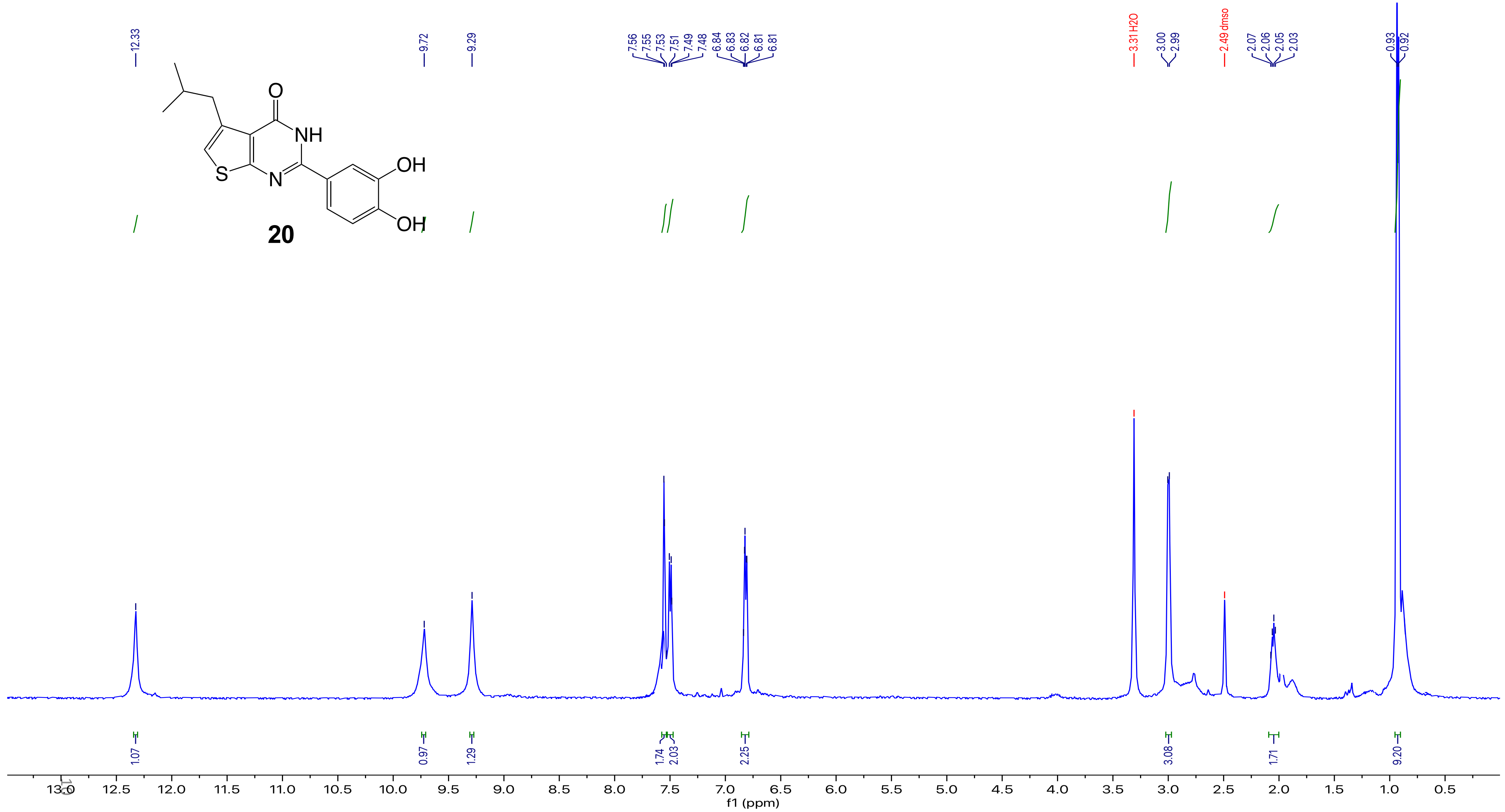
12.33
9.72
9.29
7.56
7.55
7.53
7.51
7.49
7.48
6.84
6.83
6.82
6.81
6.81
3.31 H2O
3.00
2.99
2.49 dmso
2.07
2.06
2.05
2.03
0.93
0.92
O
NH
OH
OH
S
N
20
1.07
0.97
1.29
1.74
2.03
2.25
3.08
1.71
9.20
13.0
12.5
12.0
11.5
11.0
10.5
10.0
9.5
9.0
8.5
8.0
7.5
7.0
6.5
6.0
5.5
5.0
4.5
4.0
3.5
3.0
2.5
2.0
1.5
1.0
0.5
f1 (ppm)

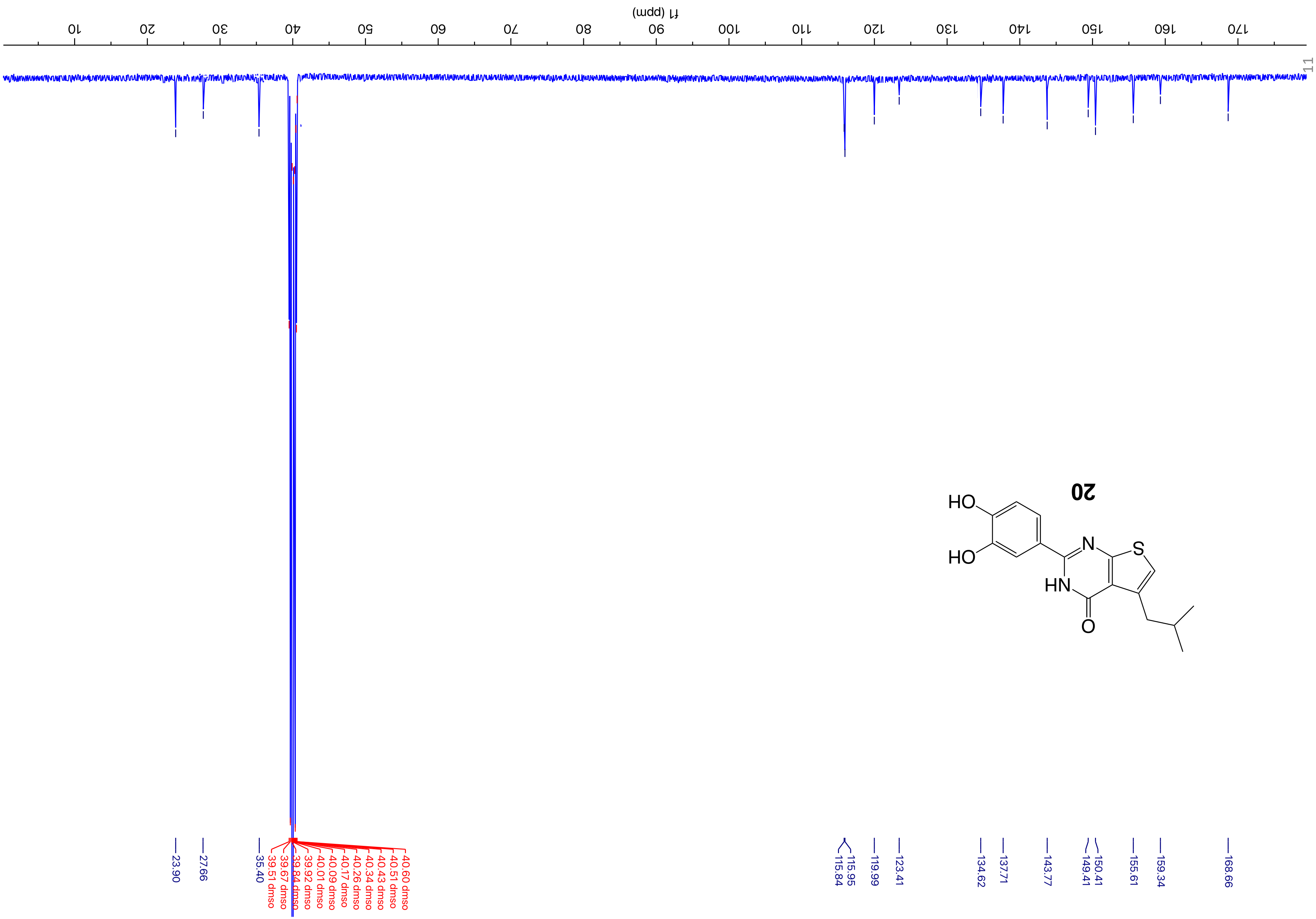
f1 (ppm)
10
20
30
40
50
60
70
80
90
100
110
120
130
140
150
160
170
23.90
27.66
35.40
39.51 dmso
39.67 dmso
39.84 dmso
39.92 dmso
40.01 dmso
40.09 dmso
40.17 dmso
40.26 dmso
40.34 dmso
40.43 dmso
40.51 dmso
40.60 dmso
115.84
115.95
119.99
123.41
134.62
137.71
143.77
149.41
150.41
155.61
159.34
168.66
HO
HO
N
S
HN
O
20

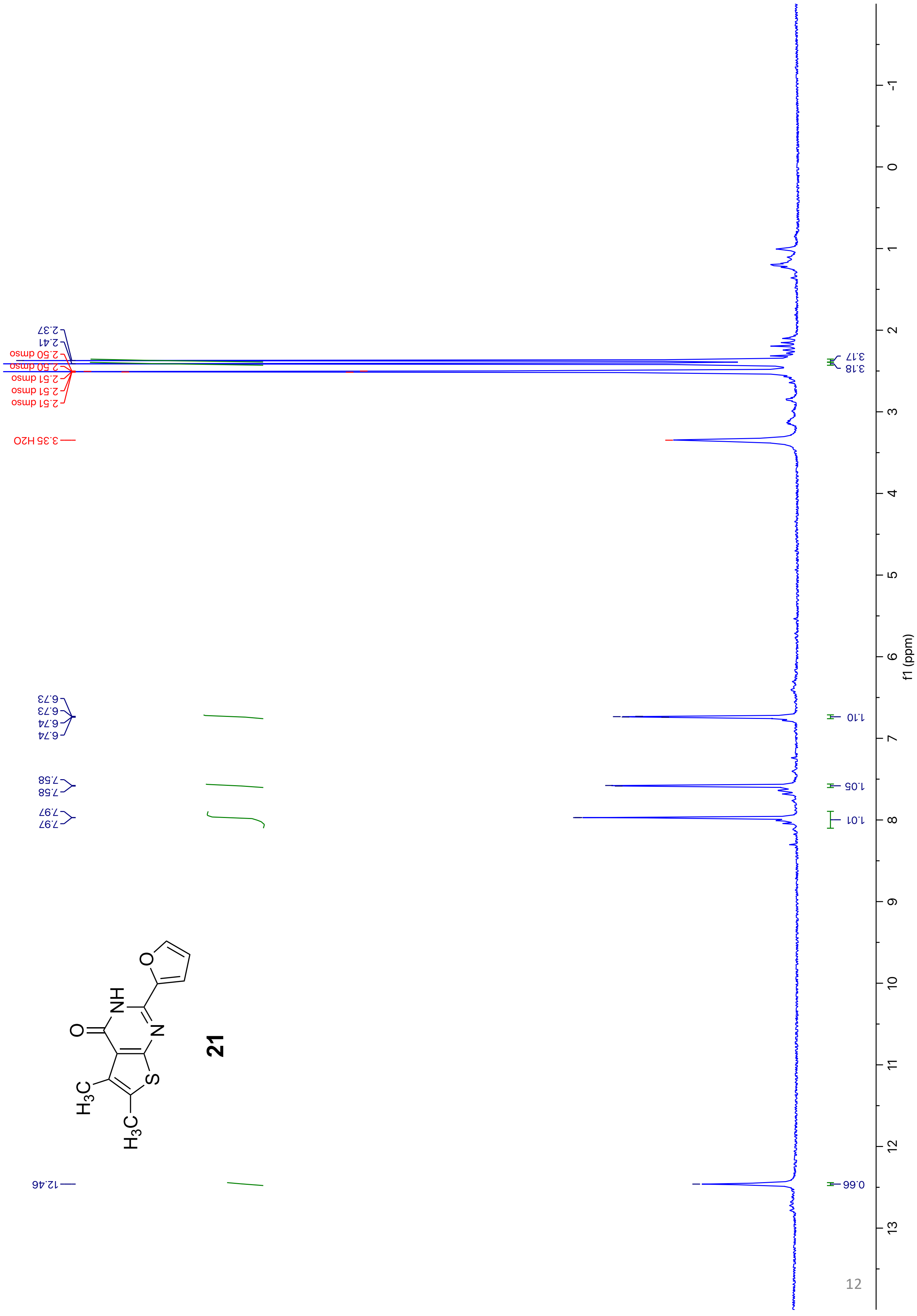
f1 (ppm)
12.46
7.97
7.97
7.58
7.58
6.74
6.74
6.73
6.73
3.35 H2O
2.51 dmso
2.51 dmso
2.51 dmso
2.50 dmso
2.50 dmso
2.41
2.37
0.66
1.01
1.05
1.10
3.18
3.17
H3C
H3C
S
N
NH
O
O

21

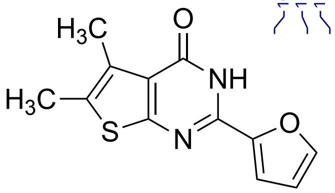

21

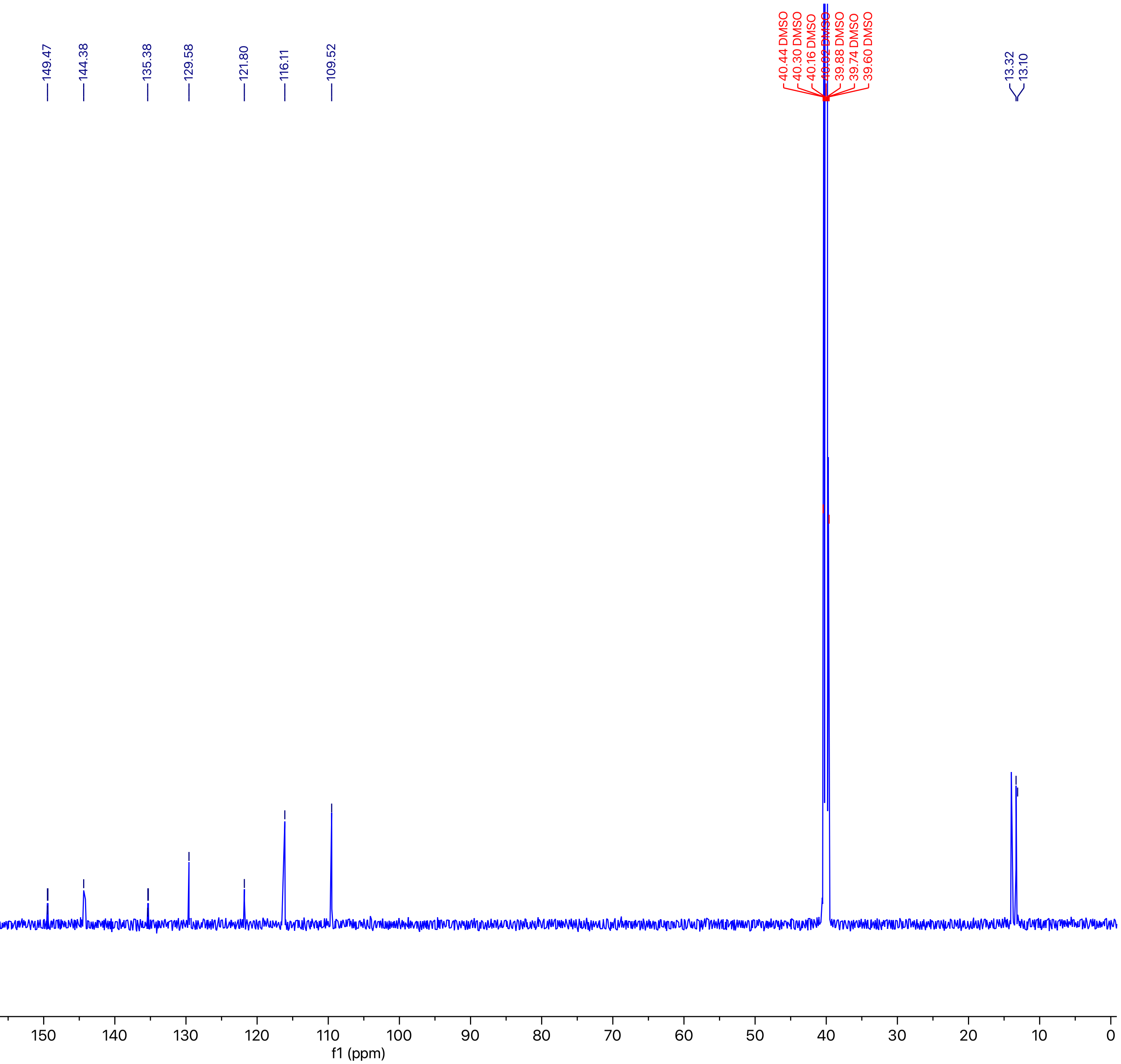
162.53
158.94
156.49
149.47
144.38
135.38
129.58
121.80
116.11
109.52
40.44 DMSO
40.30 DMSO
40.16 DMSO
40.02 DMSO
39.88 DMSO
39.74 DMSO
39.60 DMSO
13.32
13.10
210
200
190
180
170
160
150
140
130
120
110
100
90
80
70
60
50
40
30
20
10
0
f1 (ppm)

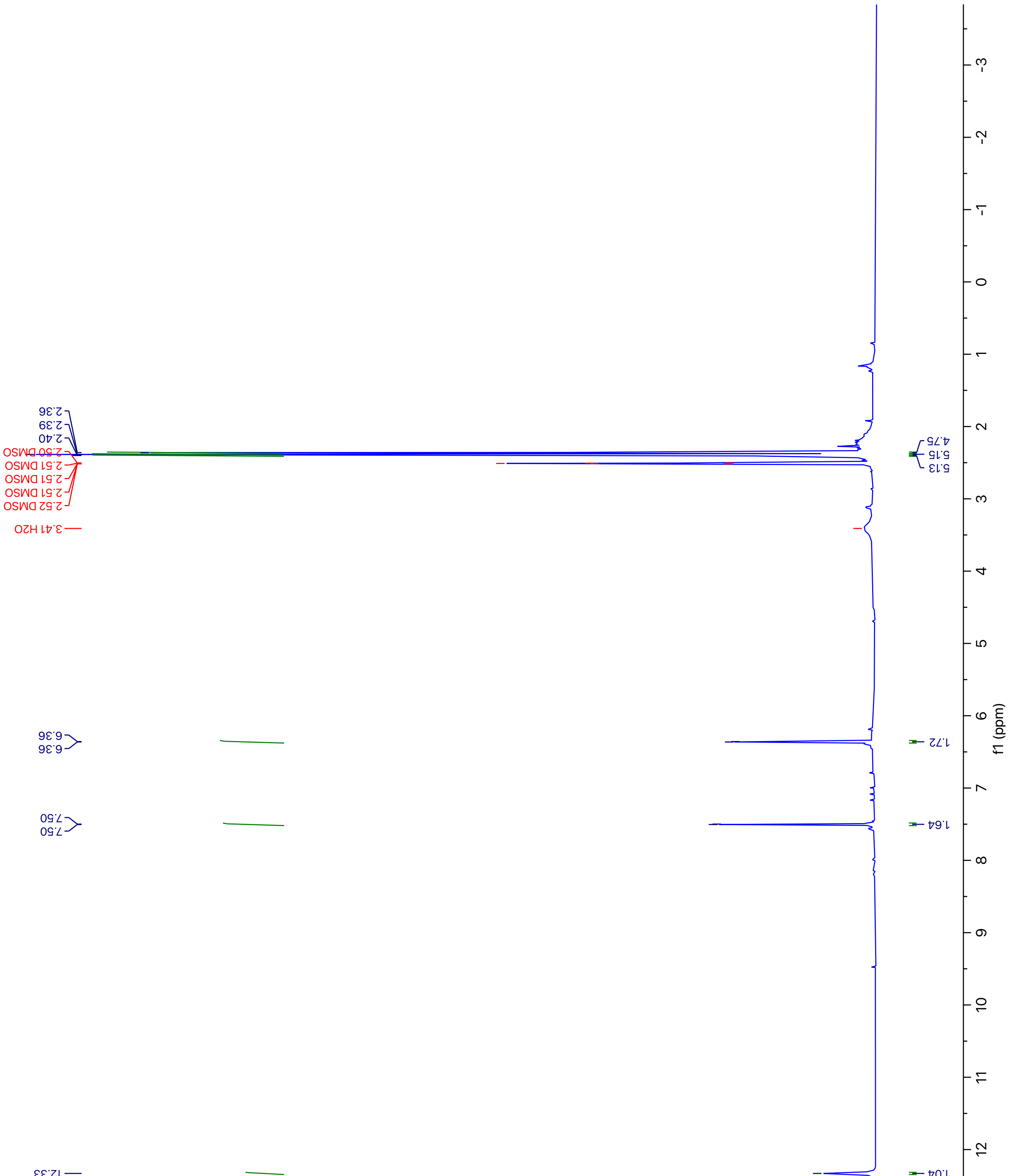
12.33
7.50
7.50
6.36
6.36
3.41 H2O
2.52 DMSO
2.51 DMSO
2.51 DMSO
2.51 DMSO
2.50 DMSO
2.40
2.39
2.36
1.04
1.64
1.72
5.13
5.15
4.75
f1 (ppm)

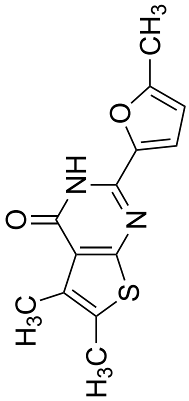
O
NH
N
S
O
CH3
H3C
H3C

22

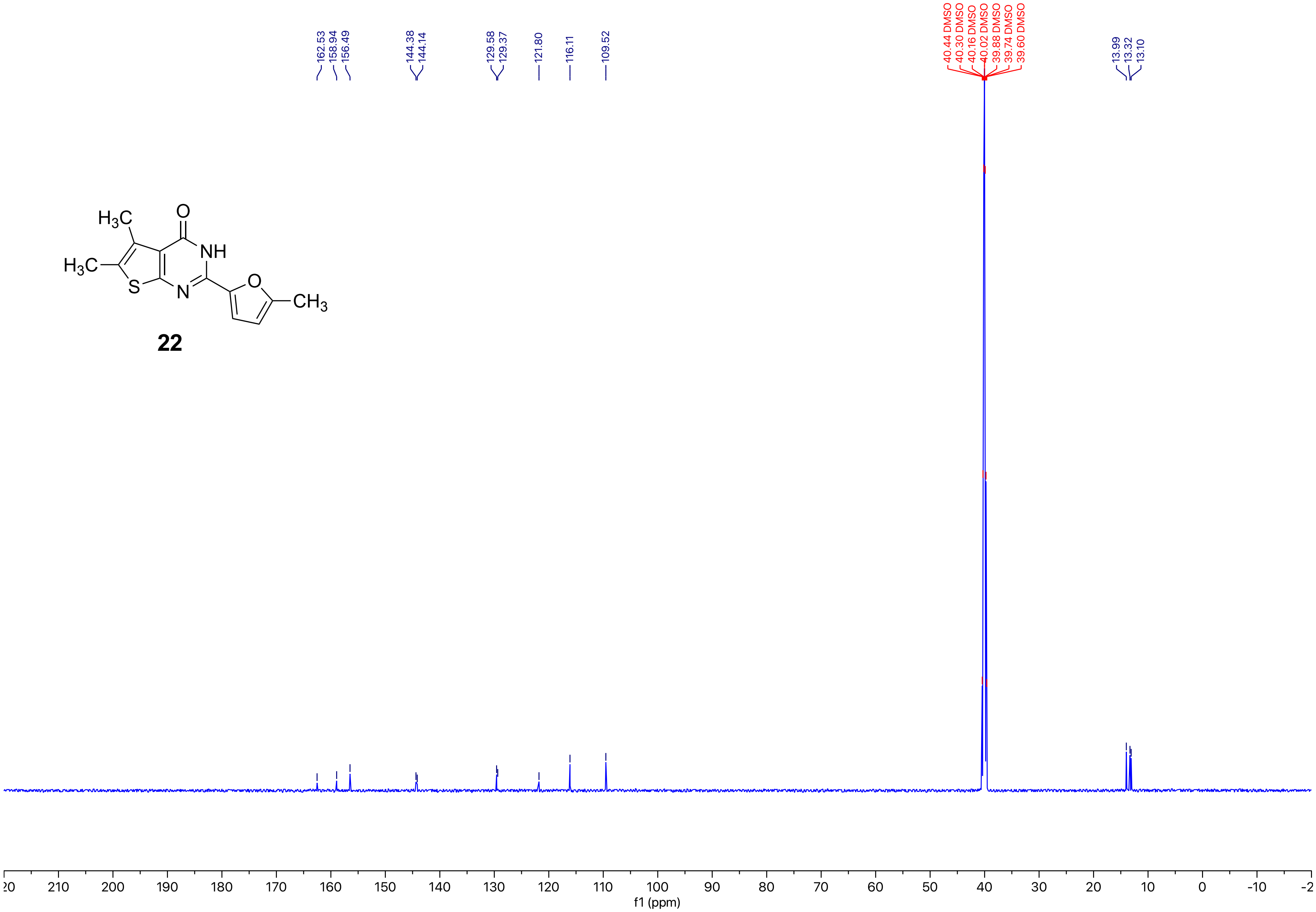
H3C
H3C
O
NH
S
N
O
CH3
22
162.53
158.94
156.49
144.38
144.14
129.58
129.37
121.80
116.11
109.52
40.44 DMSO
40.30 DMSO
40.16 DMSO
40.02 DMSO
39.88 DMSO
39.74 DMSO
39.60 DMSO
13.99
13.32
13.10
f1 (ppm)

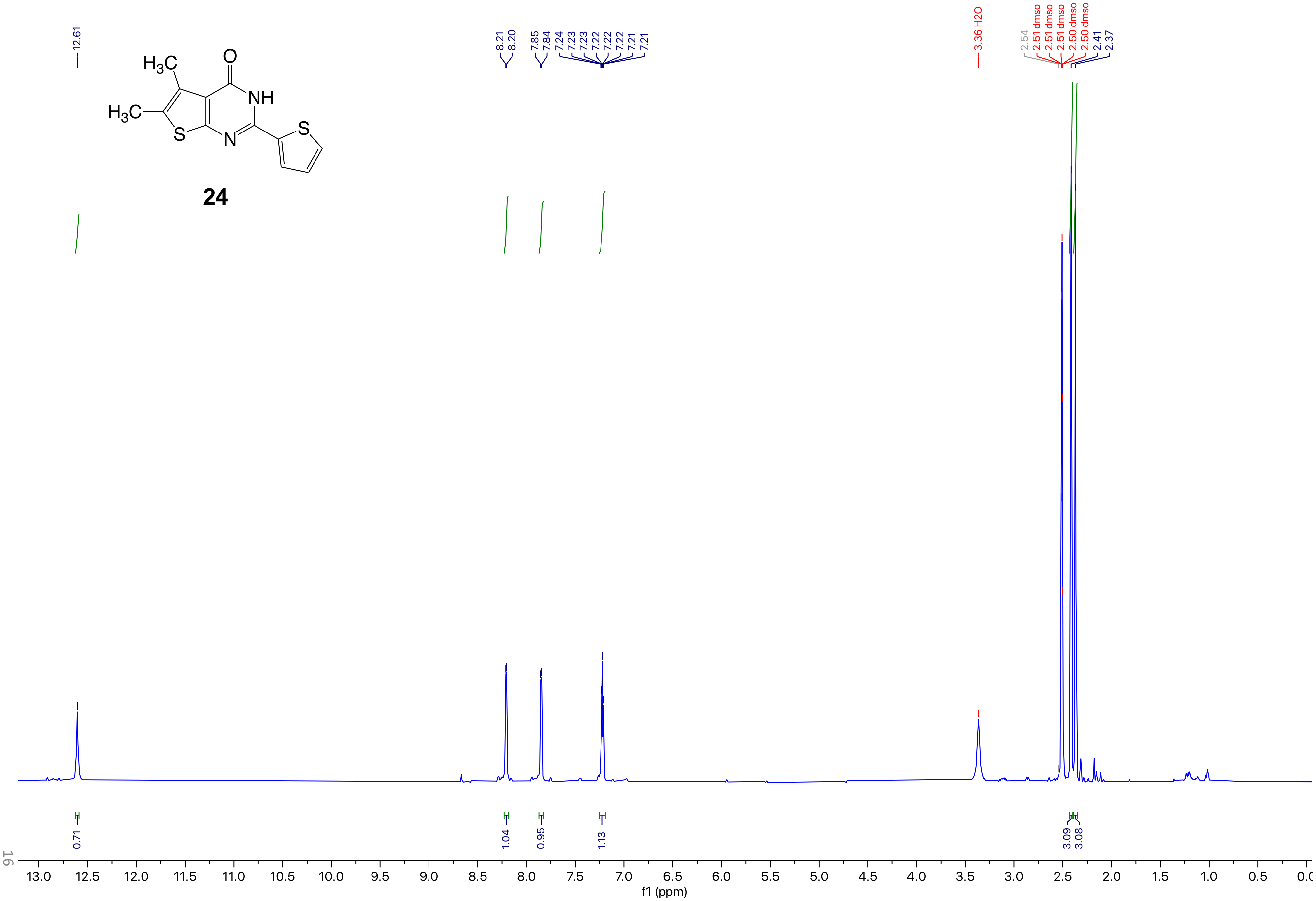
12.61
8.21
8.20
7.85
7.84
7.24
7.23
7.23
7.22
7.22
7.22
7.21
7.21
3.36 H2O
2.54
2.51 dmso
2.51 dmso
2.51 dmso
2.50 dmso
2.50 dmso
2.41
2.37
0.71
1.04
0.95
1.13
3.09
3.08
f1 (ppm)
H3C
H3C
O
NH
S
N
S

24

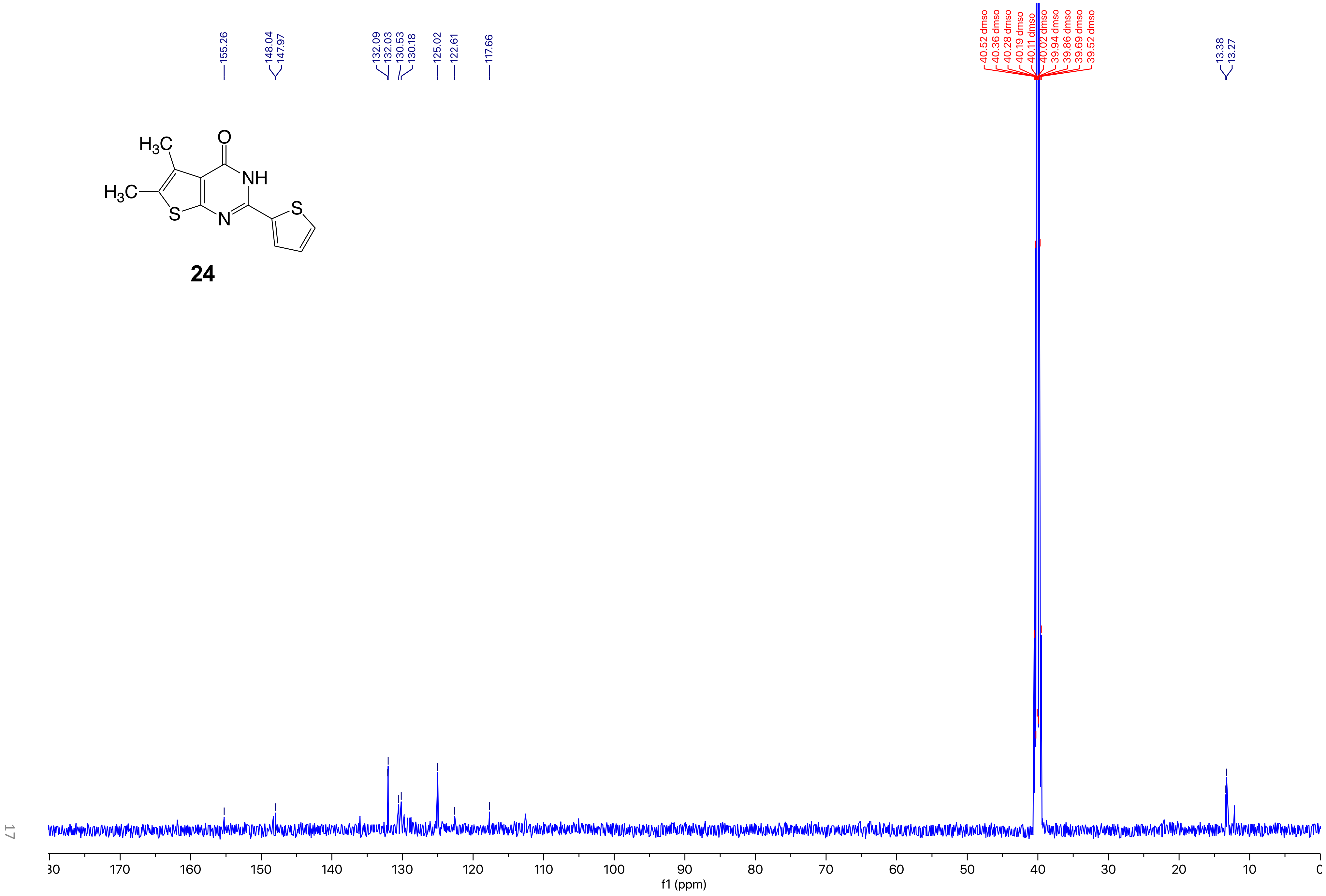
155.26
148.04
147.97
132.09
132.03
130.53
130.18
125.02
122.61
117.66
40.52 dmso
40.36 dmso
40.28 dmso
40.19 dmso
40.11 dmso
40.02 dmso
39.94 dmso
39.86 dmso
39.69 dmso
39.52 dmso
13.38
13.27
f1 (ppm)

24

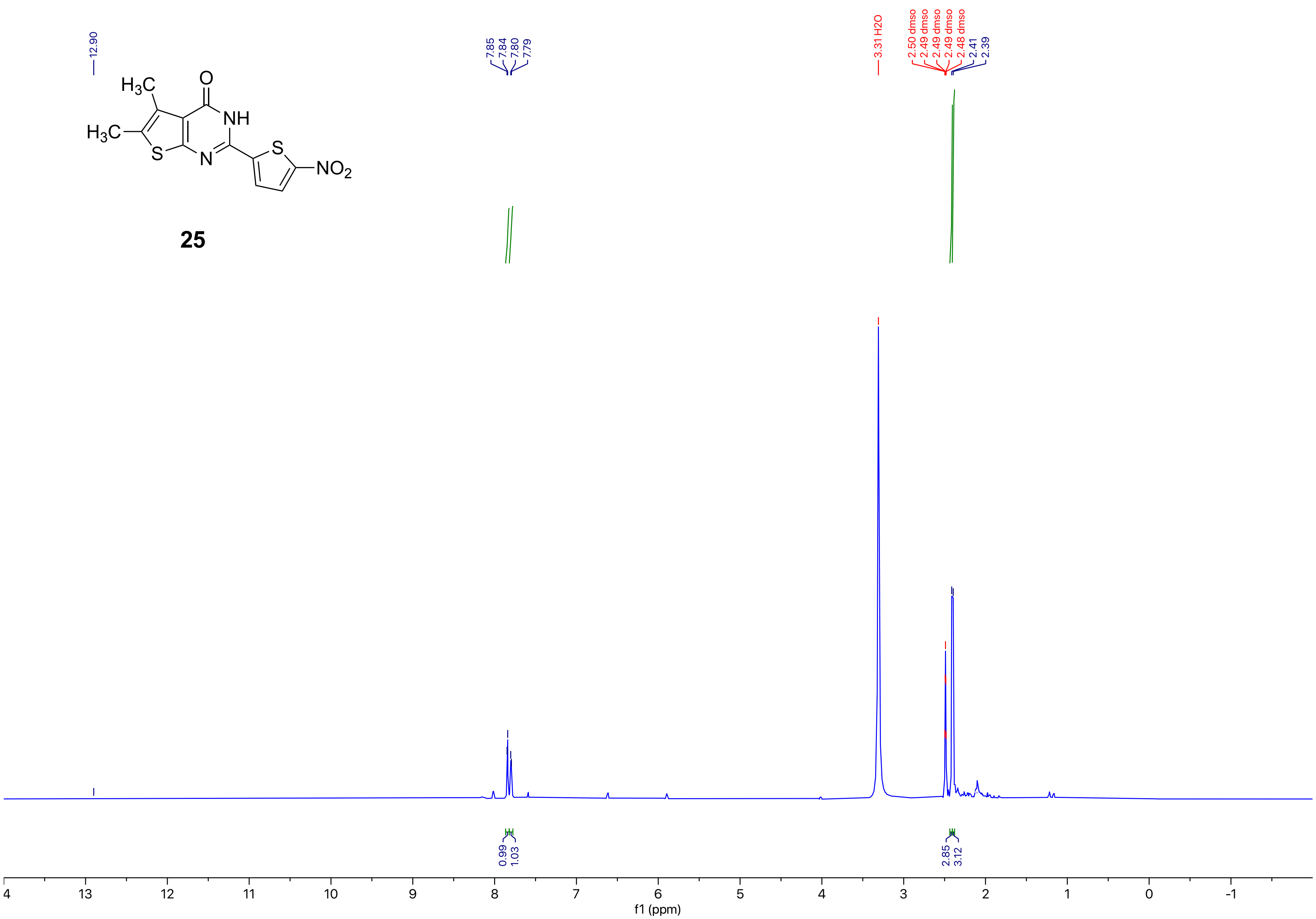

12.90
7.85
7.84
7.80
7.79
3.31 H2O
2.50 dmso
2.49 dmso
2.49 dmso
2.49 dmso
2.48 dmso
2.41
2.39
0.99
1.03
2.85
3.12
f1 (ppm)

25

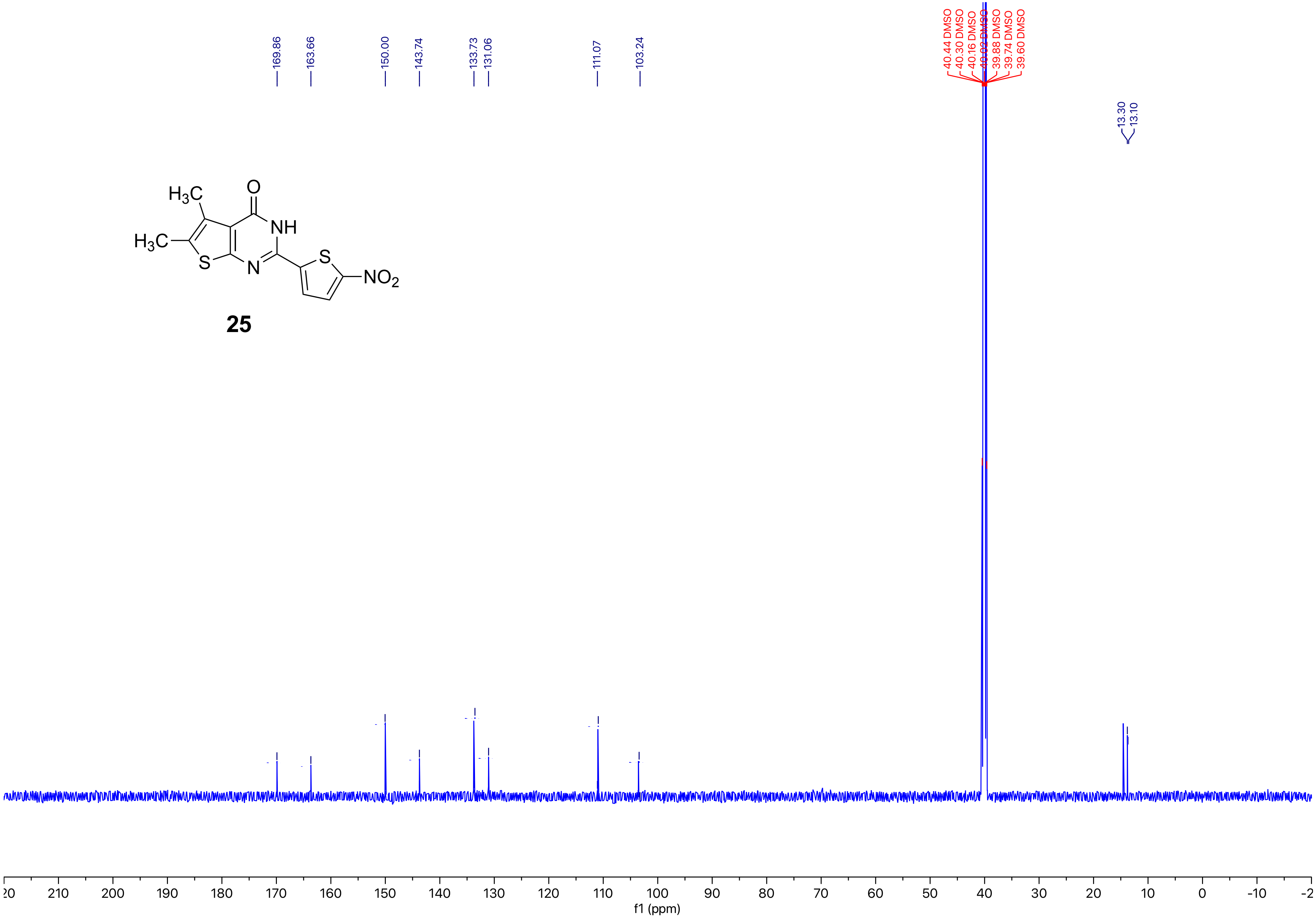
169.86
163.66
150.00
143.74
133.73
131.06
111.07
103.24
40.44 DMSO
40.30 DMSO
40.16 DMSO
40.02 DMSO
39.88 DMSO
39.74 DMSO
39.60 DMSO
13.30
13.10
210 200 190 180 170 160 150 140 130 120 110 100 90 80 70 60 50 40 30 20 10 0 -10
f1 (ppm)

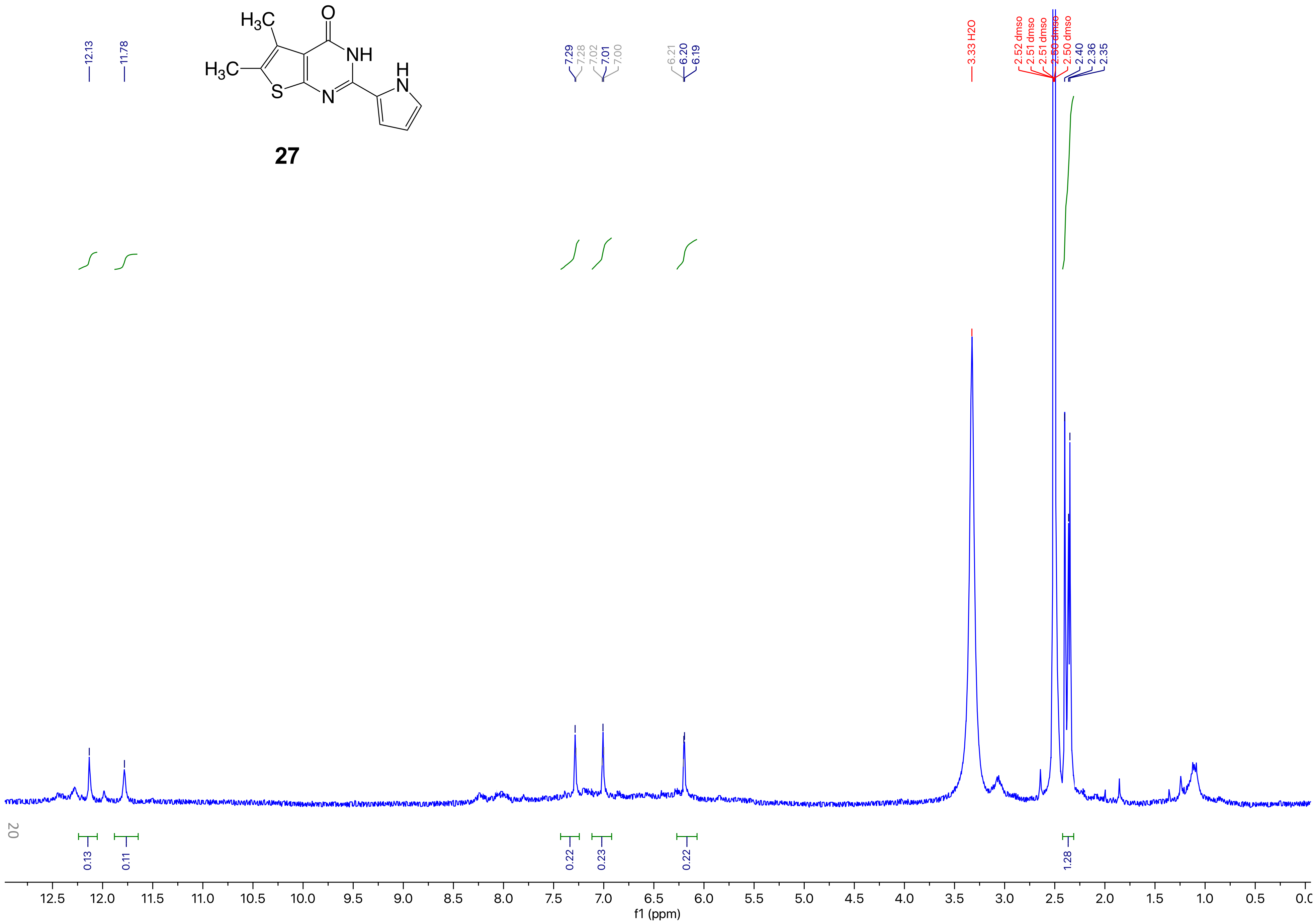
H3C
H3C
O
NH
H
N
S
N
12.13
11.78
7.29
7.28
7.02
7.01
7.00
6.21
6.20
6.19
3.33 H2O
2.52 dmso
2.51 dmso
2.51 dmso
2.50 dmso
2.50 dmso
2.40
2.36
2.35
0.13
0.11
0.22
0.23
0.22
1.28
f1 (ppm)

27

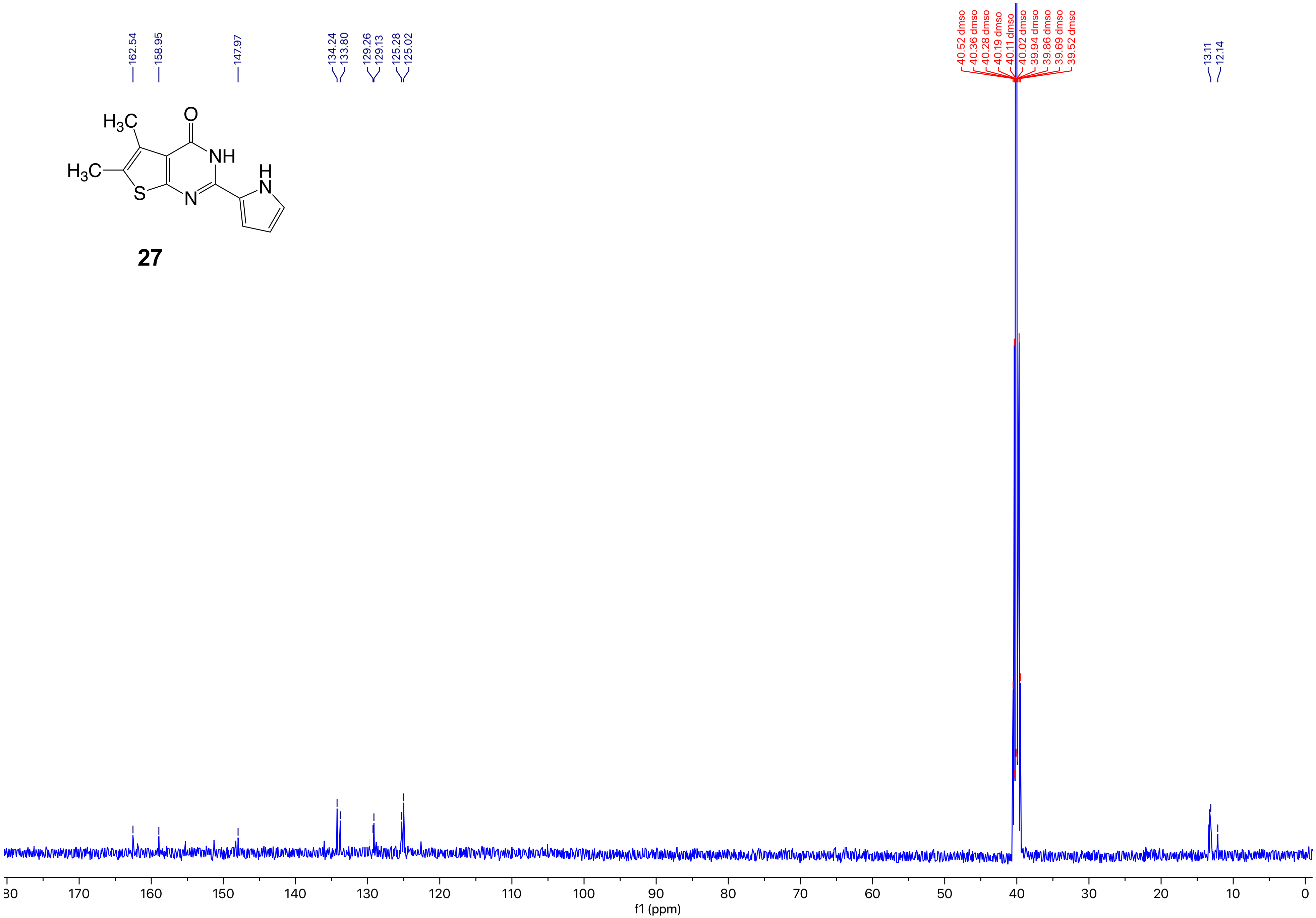

162.54
158.95
147.97
134.24
133.80
129.26
129.13
125.28
125.02
40.52 dmso
40.36 dmso
40.28 dmso
40.19 dmso
40.11 dmso
40.02 dmso
39.94 dmso
39.86 dmso
39.69 dmso
39.52 dmso
13.11
12.14
f1 (ppm)

27

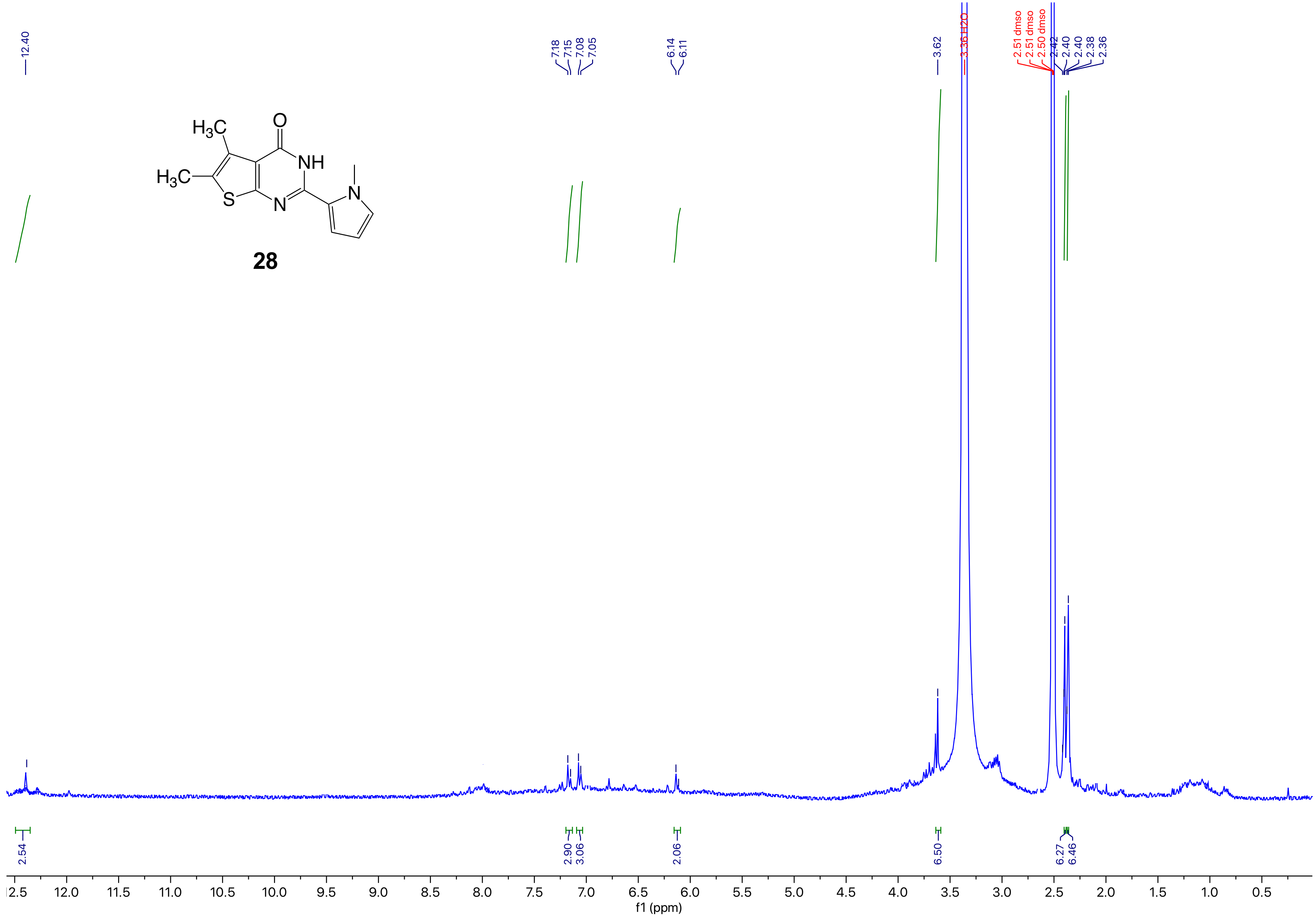
12.40
7.18
7.15
7.08
7.05
6.14
6.11
3.62
3.36 H2O
2.51 dmso
2.51 dmso
2.50 dmso
2.42
2.40
2.40
2.38
2.36
H3C
H3C
O
NH
S
N
N
2.54
2.90
3.06
2.06
6.50
6.27
6.46
12.5 12.0 11.5 11.0 10.5 10.0 9.5 9.0 8.5 8.0 7.5 7.0 6.5 6.0 5.5 5.0 4.5 4.0 3.5 3.0 2.5 2.0 1.5 1.0 0.5
f1 (ppm)

28

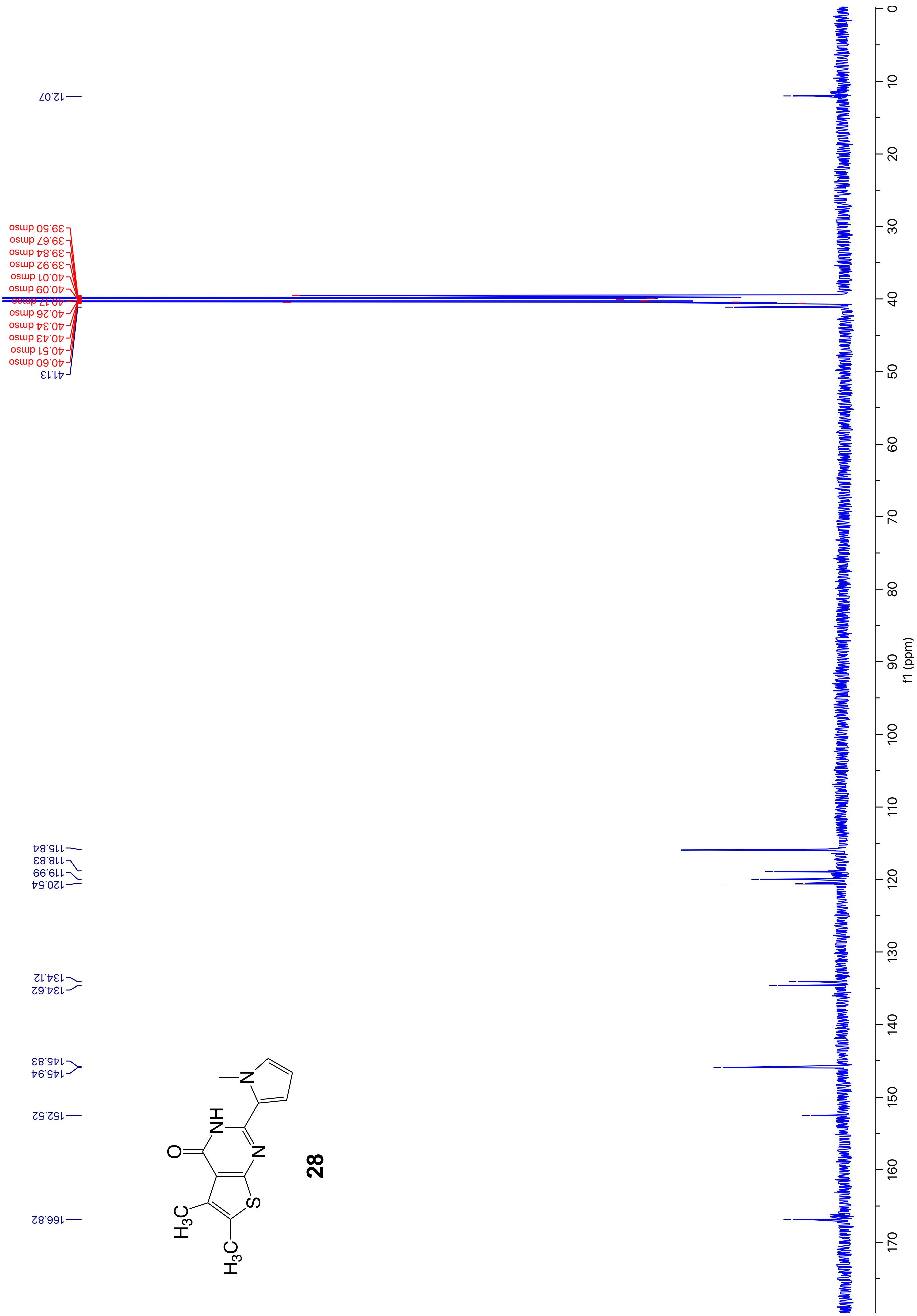
12.07
39.50 dmso
39.67 dmso
39.84 dmso
39.92 dmso
40.01 dmso
40.09 dmso
40.17 dmso
40.26 dmso
40.34 dmso
40.43 dmso
40.51 dmso
40.60 dmso
41.13
115.84
118.83
119.99
120.54
134.12
134.62
145.83
145.94
152.52
166.82
f1 (ppm)
28

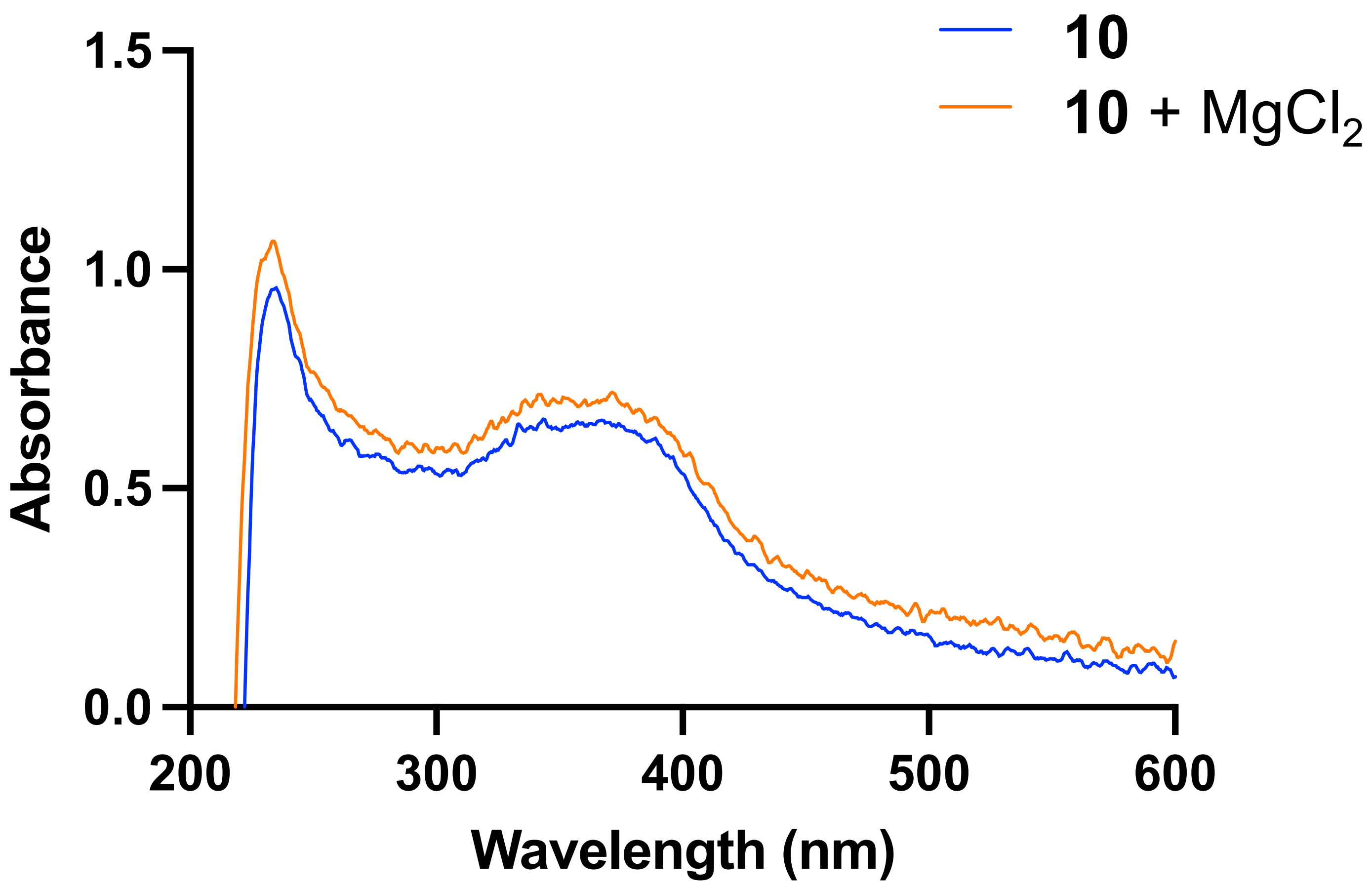
10
10 + $MgCl_2$
Absorbance
1.5
1.0
0.5
0.0
200
300
400
500
600
Wavelength (nm)

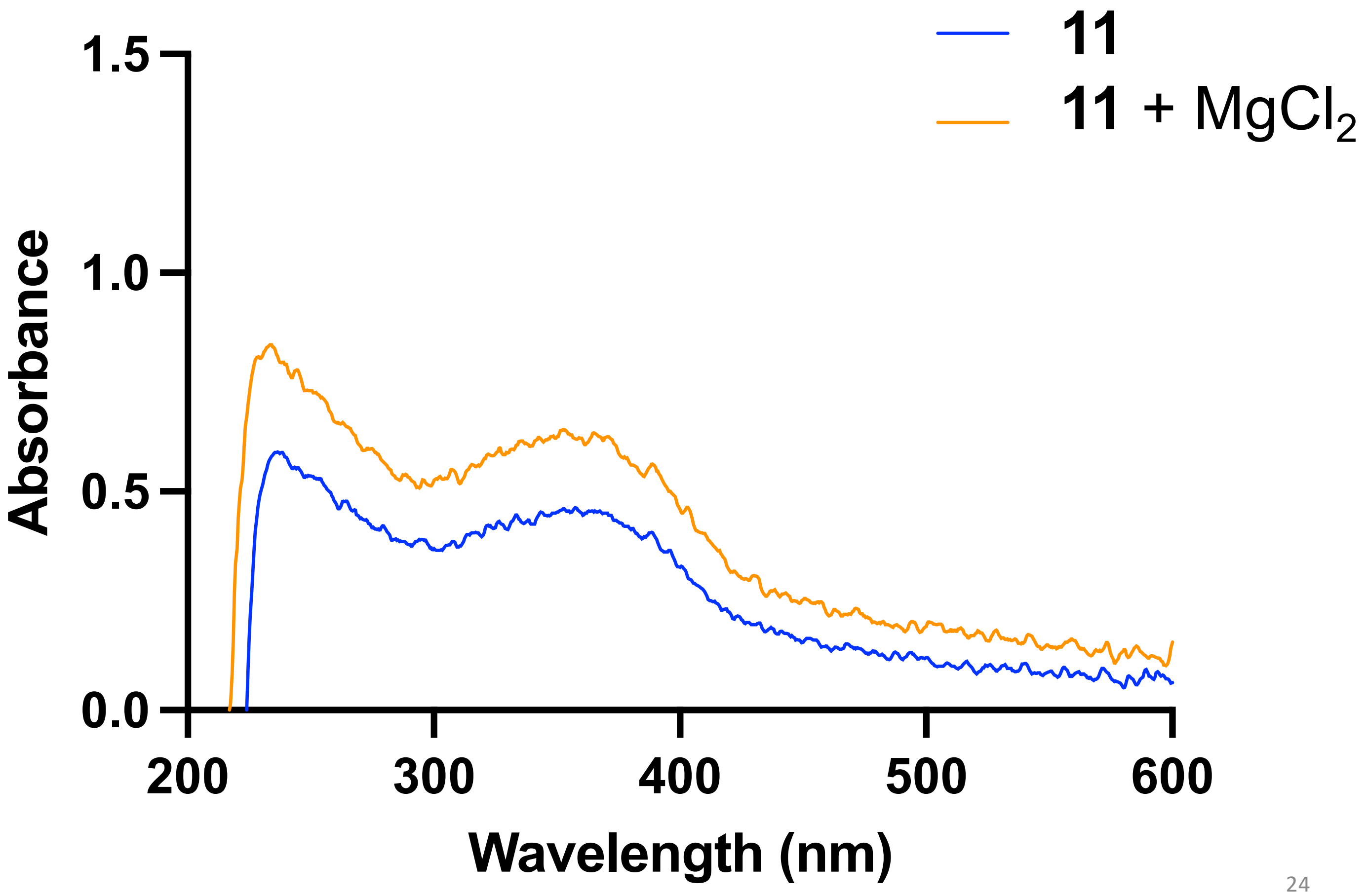
11
11 + $MgCl_2$
Absorbance
1.5
1.0
0.5
0.0
200
300
400
500
600
Wavelength (nm)

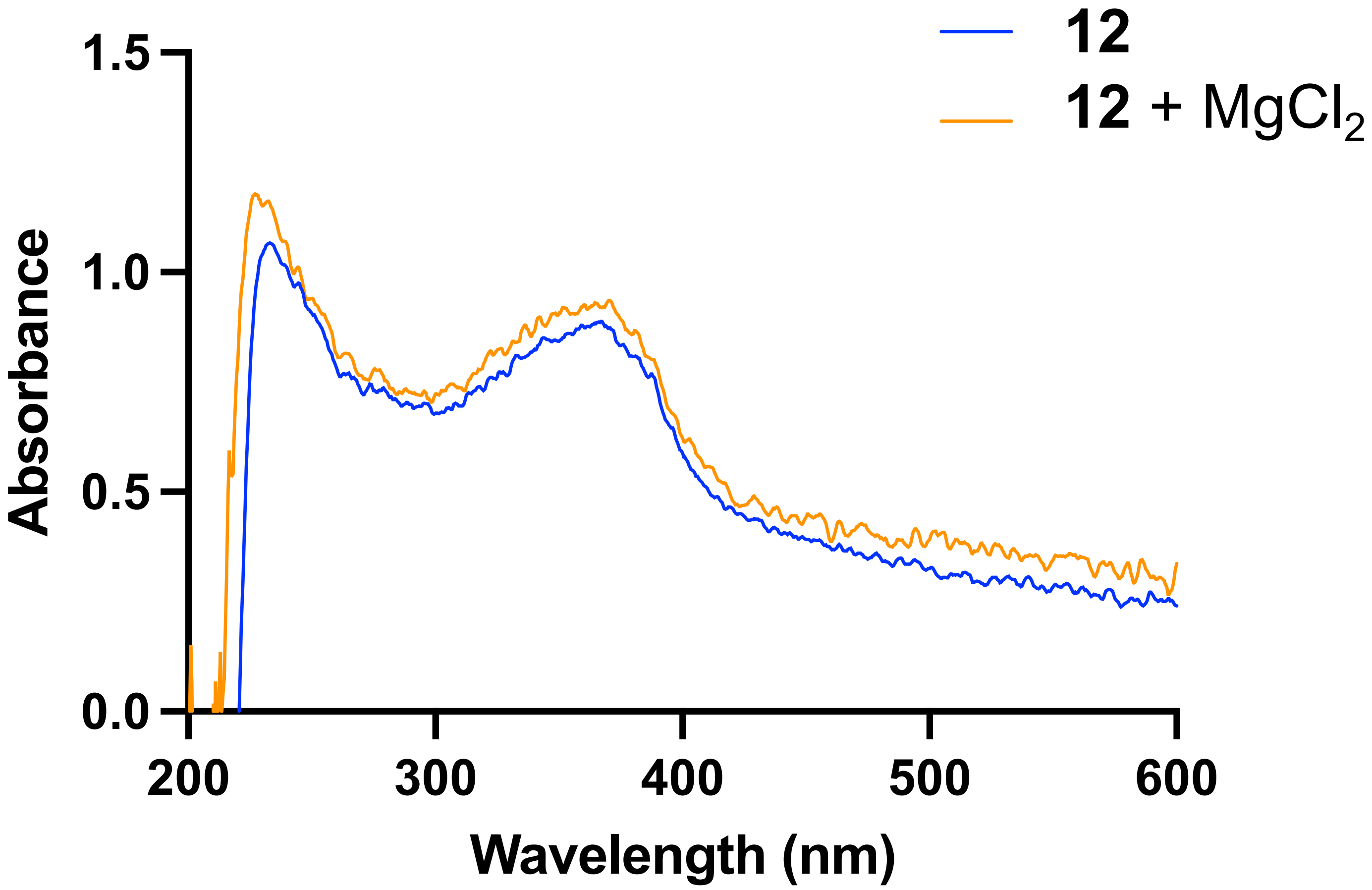
1.5
1.0
0.5
0.0
Absorbance
200
300
400
500
600
Wavelength (nm)
12
12 + MgCl2

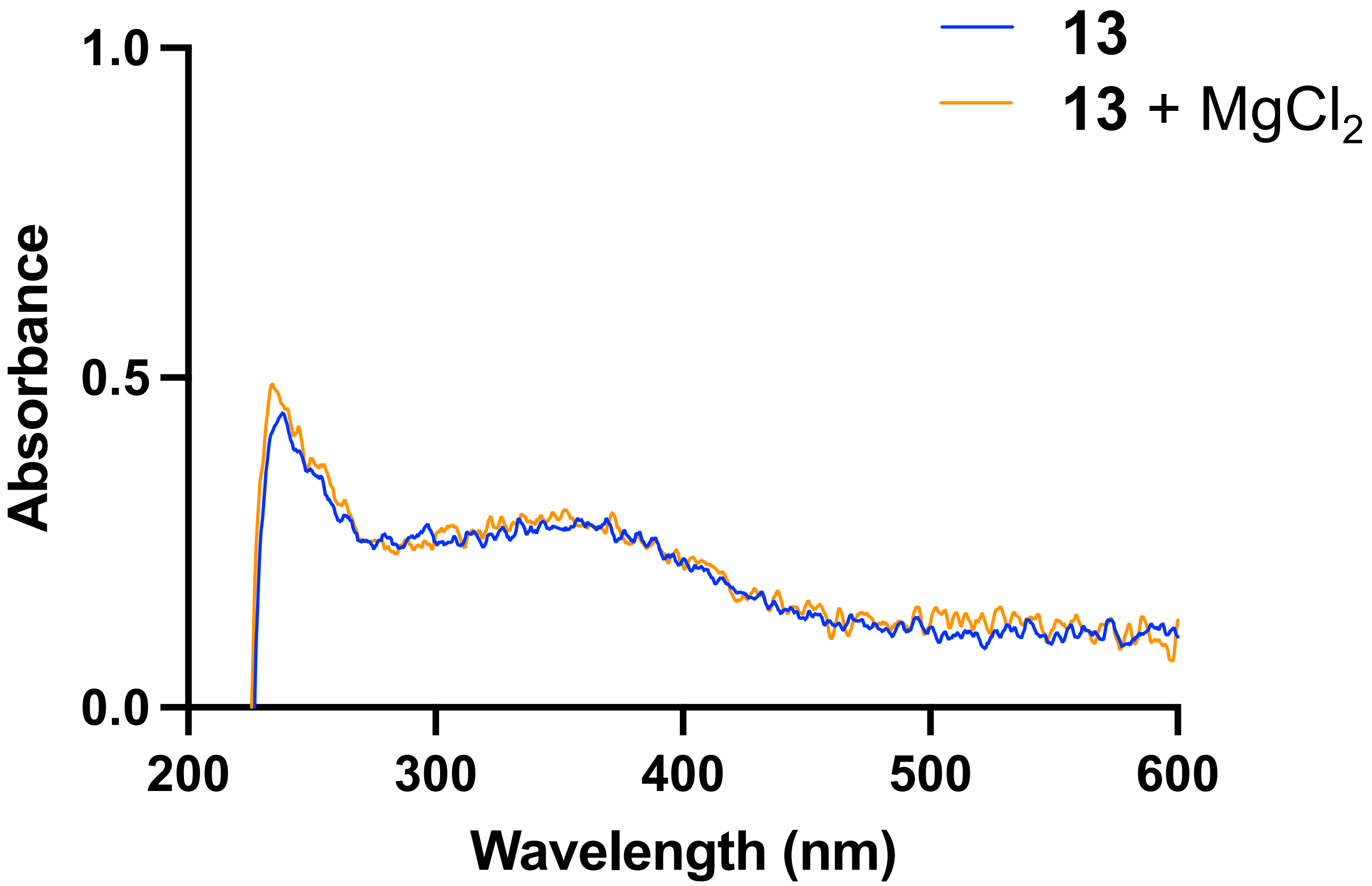
1.0
0.5
0.0
Absorbance
200
300
400
500
600
Wavelength (nm)
13
13 + MgCl2

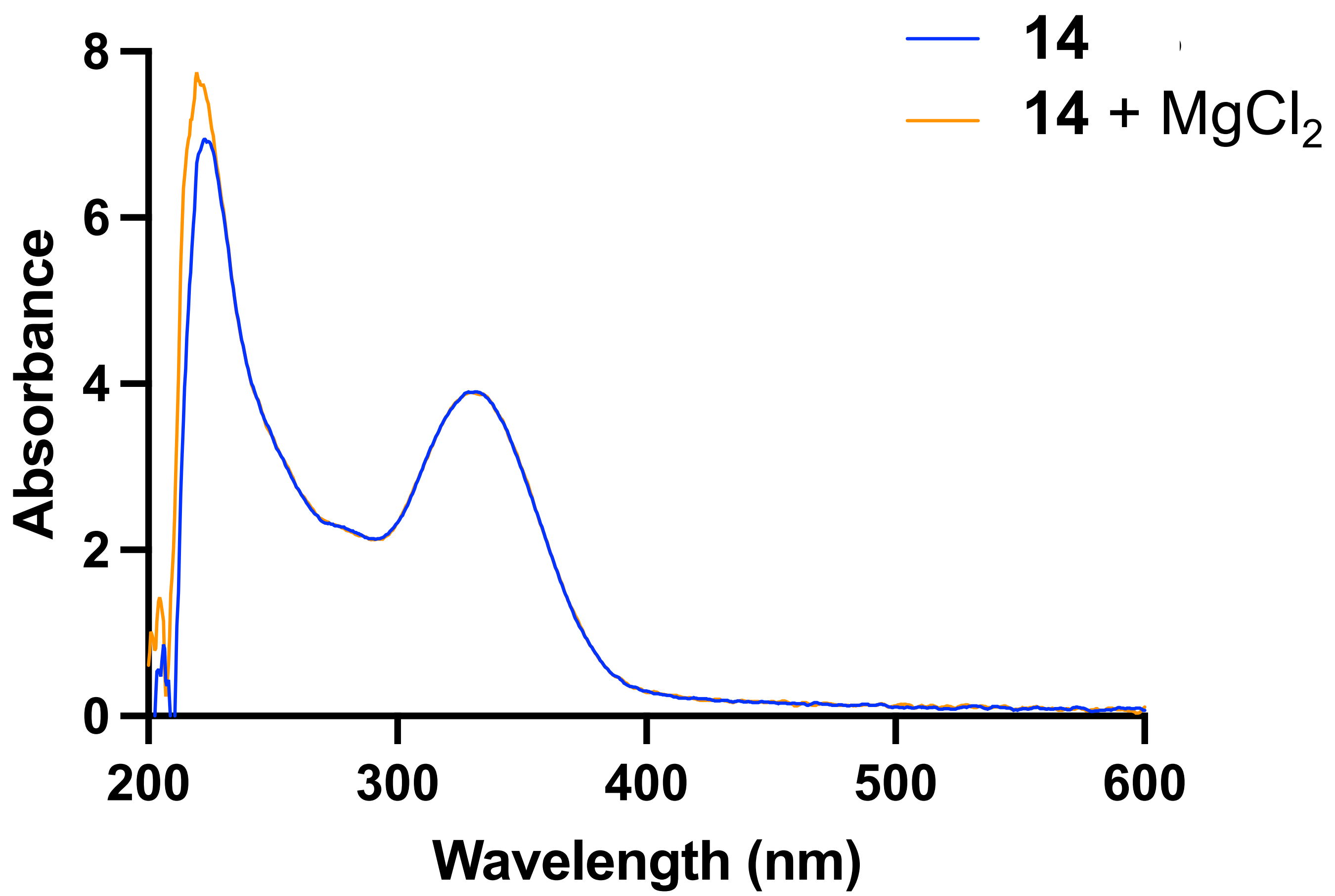

14
14 + MgCl2
Absorbance
8
6
4
2
0
200
300
400
500
600
Wavelength (nm)

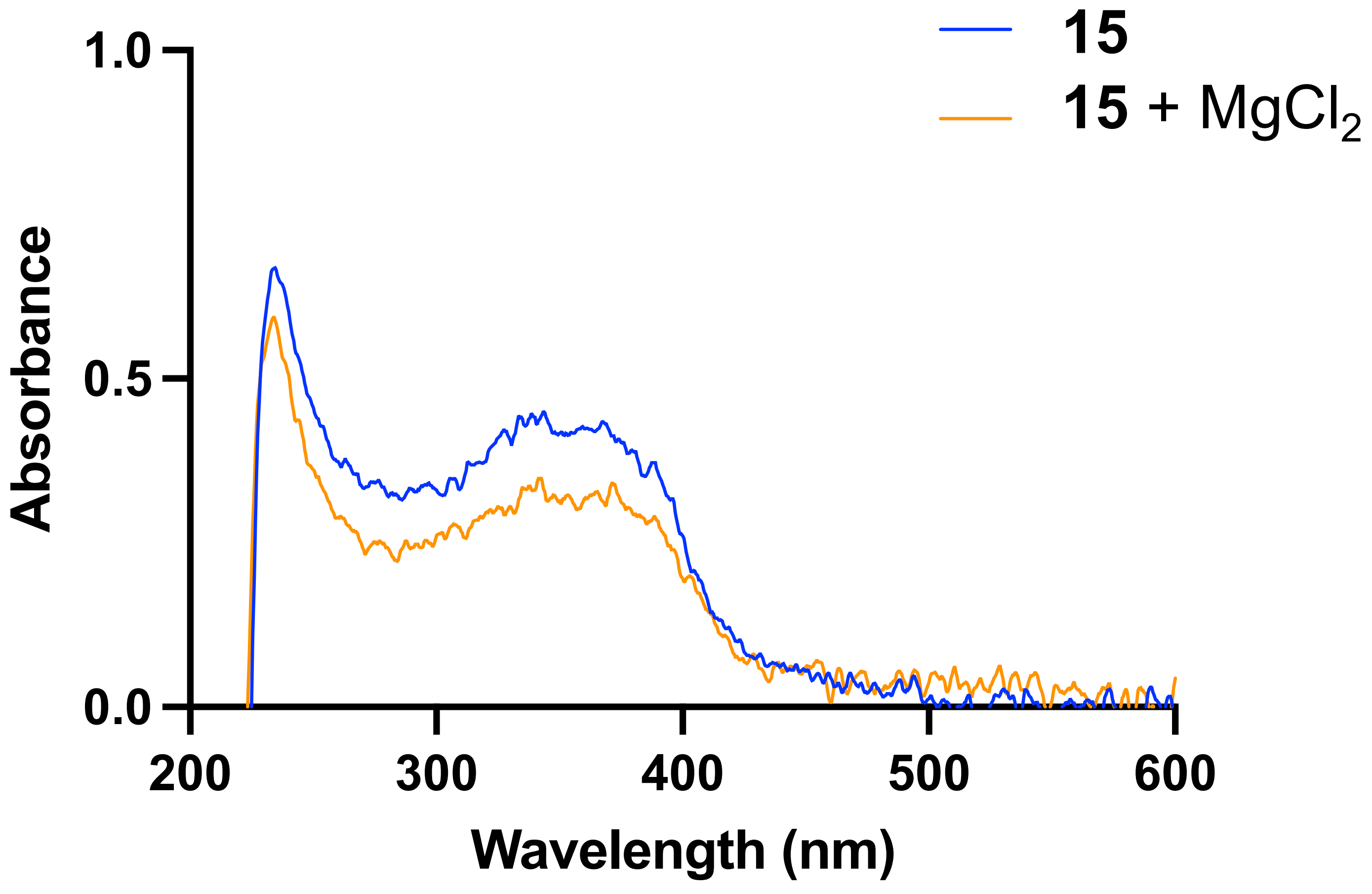

15
15 + MgCl2
Absorbance
1.0
0.5
0.0
200
300
400
500
600
Wavelength (nm)

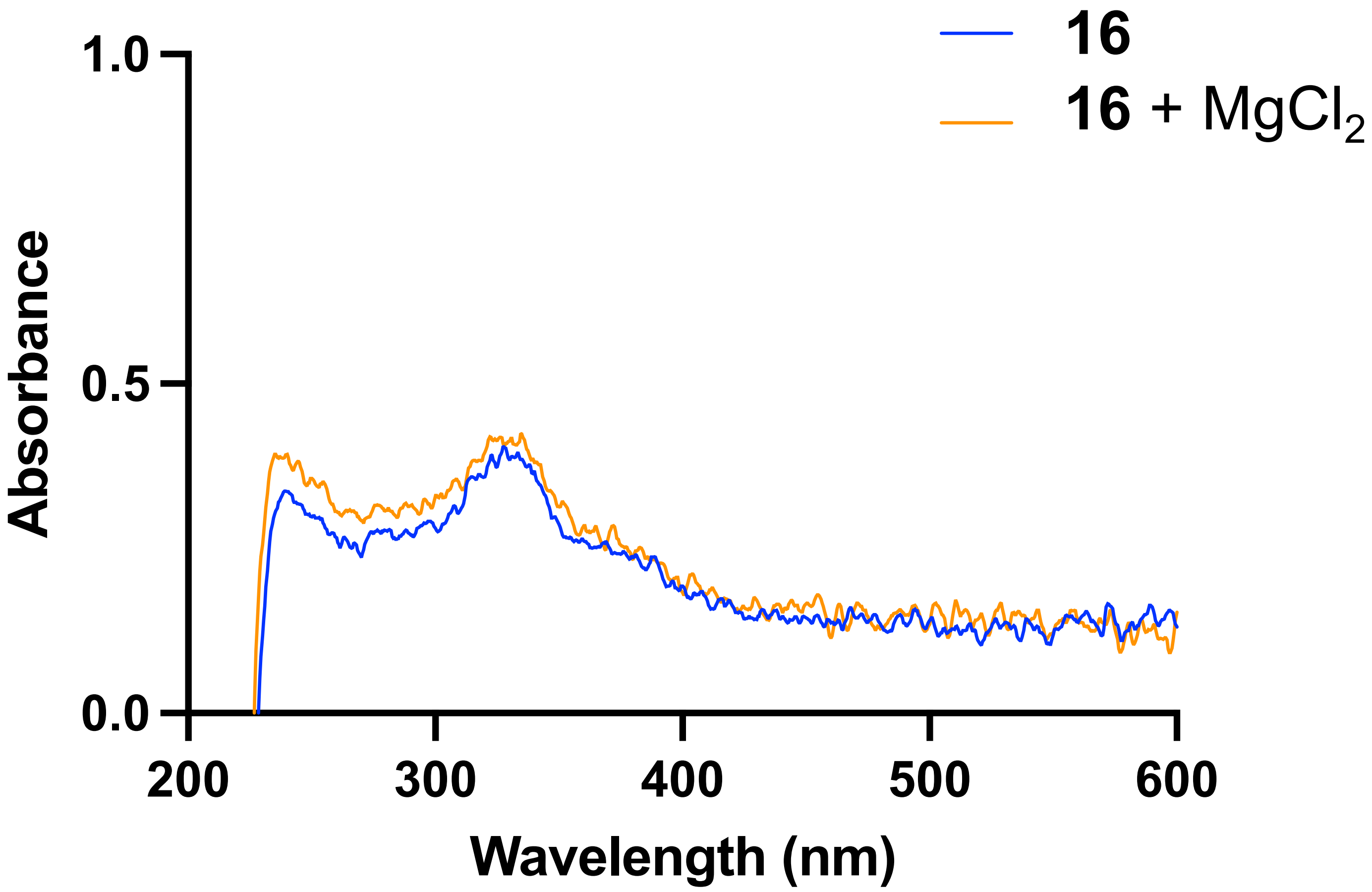
16
16 + MgCl2
1.0
0.5
0.0
Absorbance
200
300
400
500
600
Wavelength (nm)

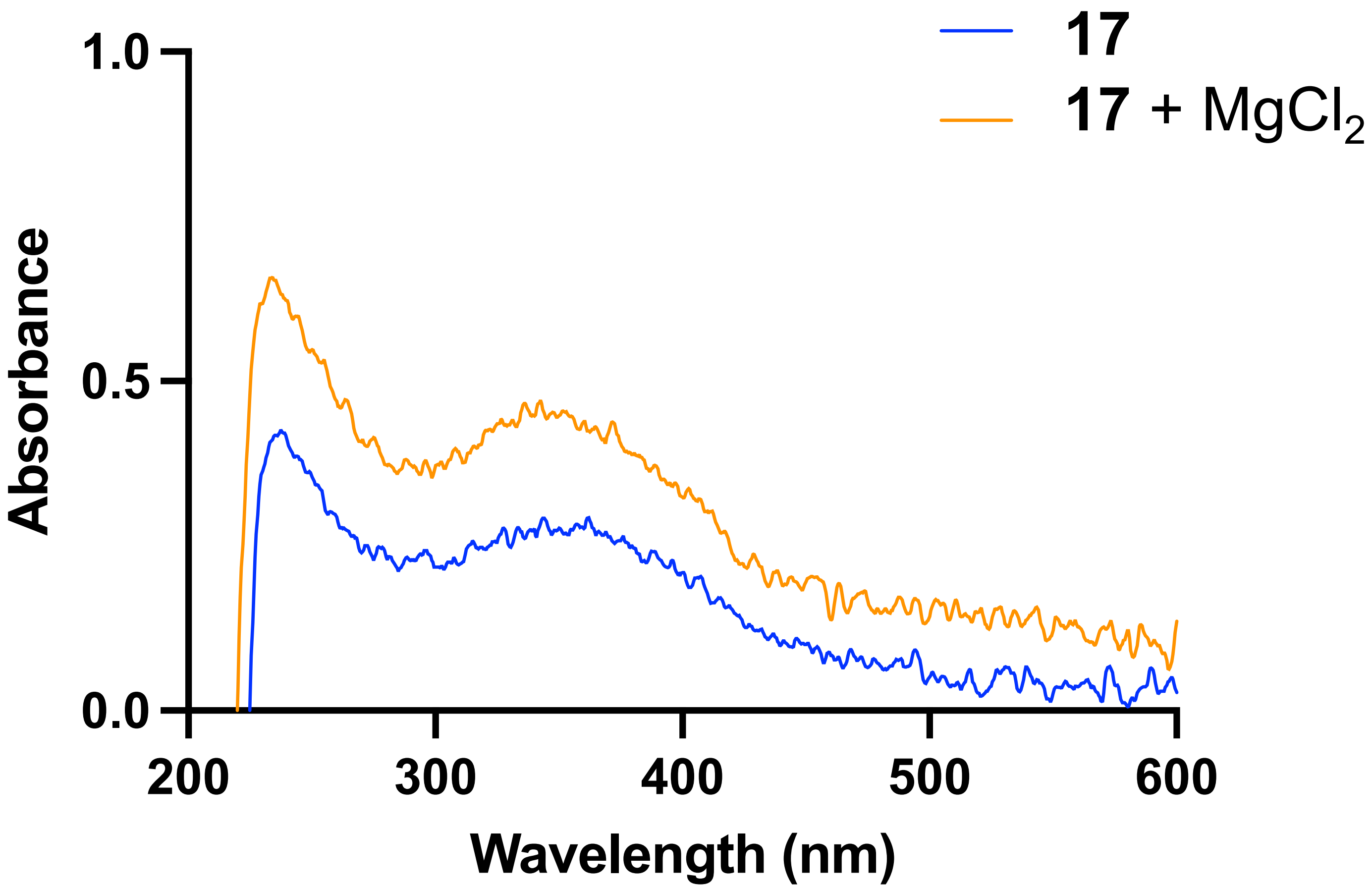
17
17 + MgCl2
1.0
0.5
0.0
Absorbance
200
300
400
500
600
Wavelength (nm)

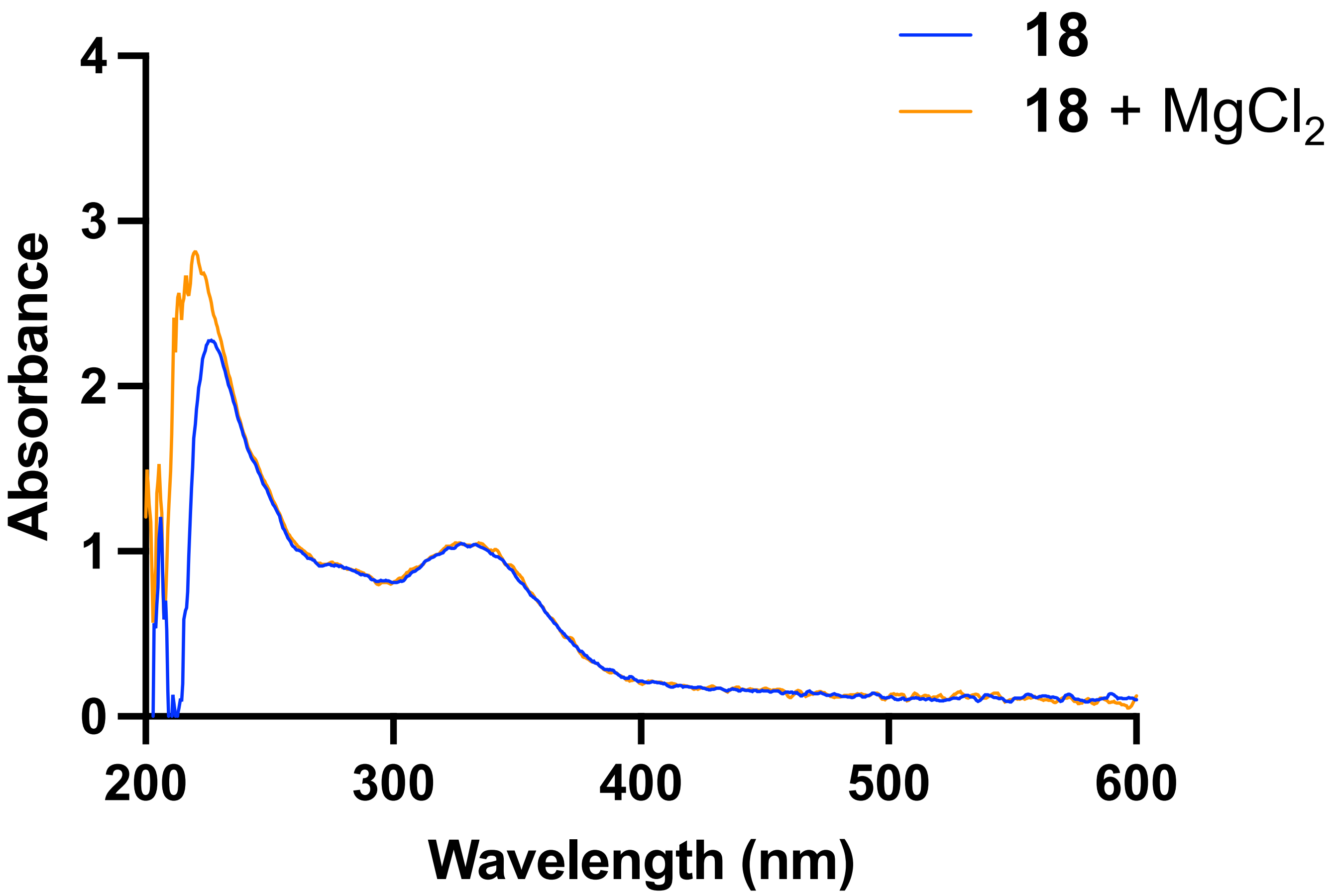
18
18 + MgCl2
Absorbance
4
3
2
1
0
200
300
400
500
600
Wavelength (nm)

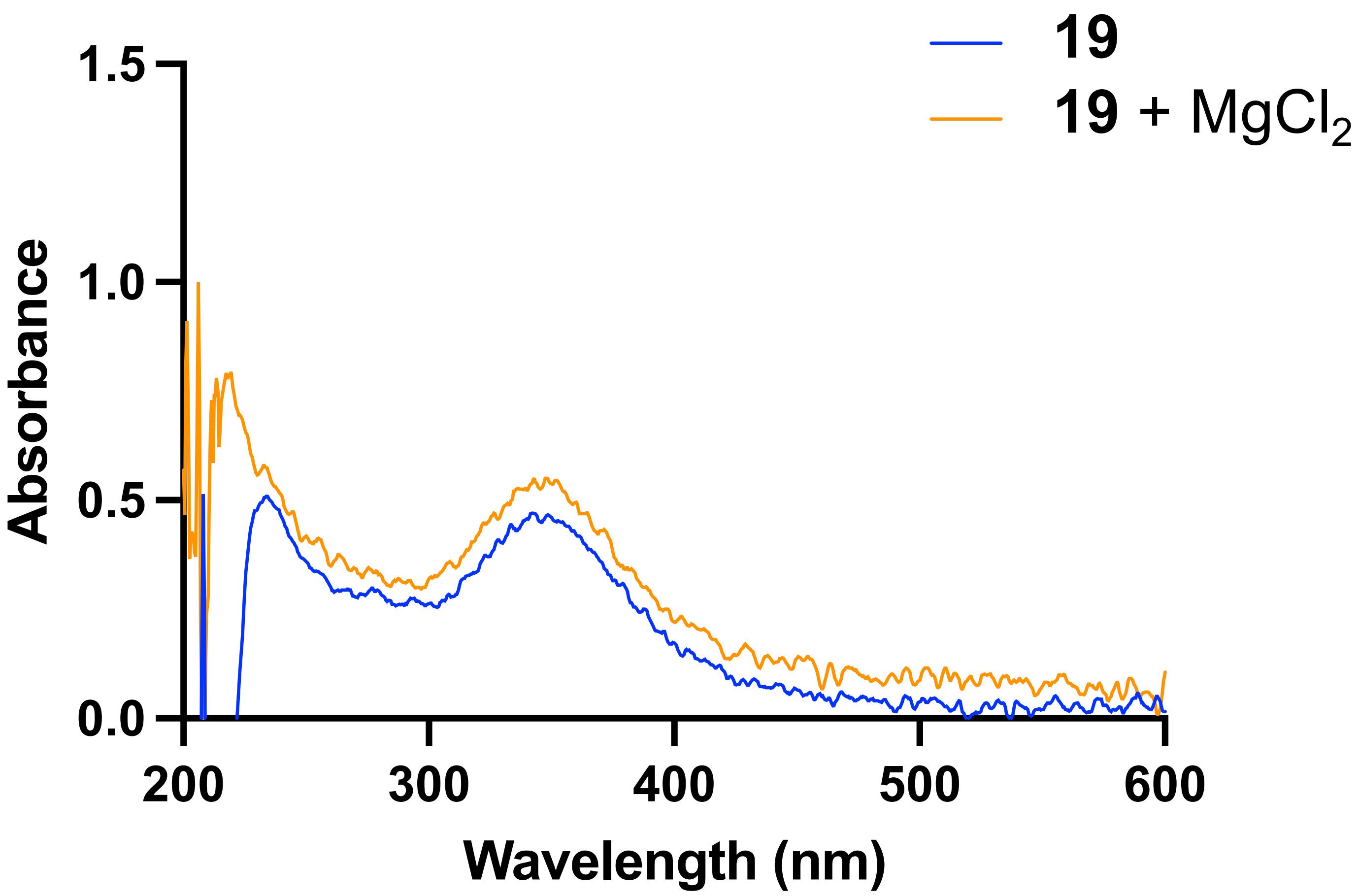
19
19 + MgCl2
Absorbance
1.5
1.0
0.5
0.0
200
300
400
500
600
Wavelength (nm)

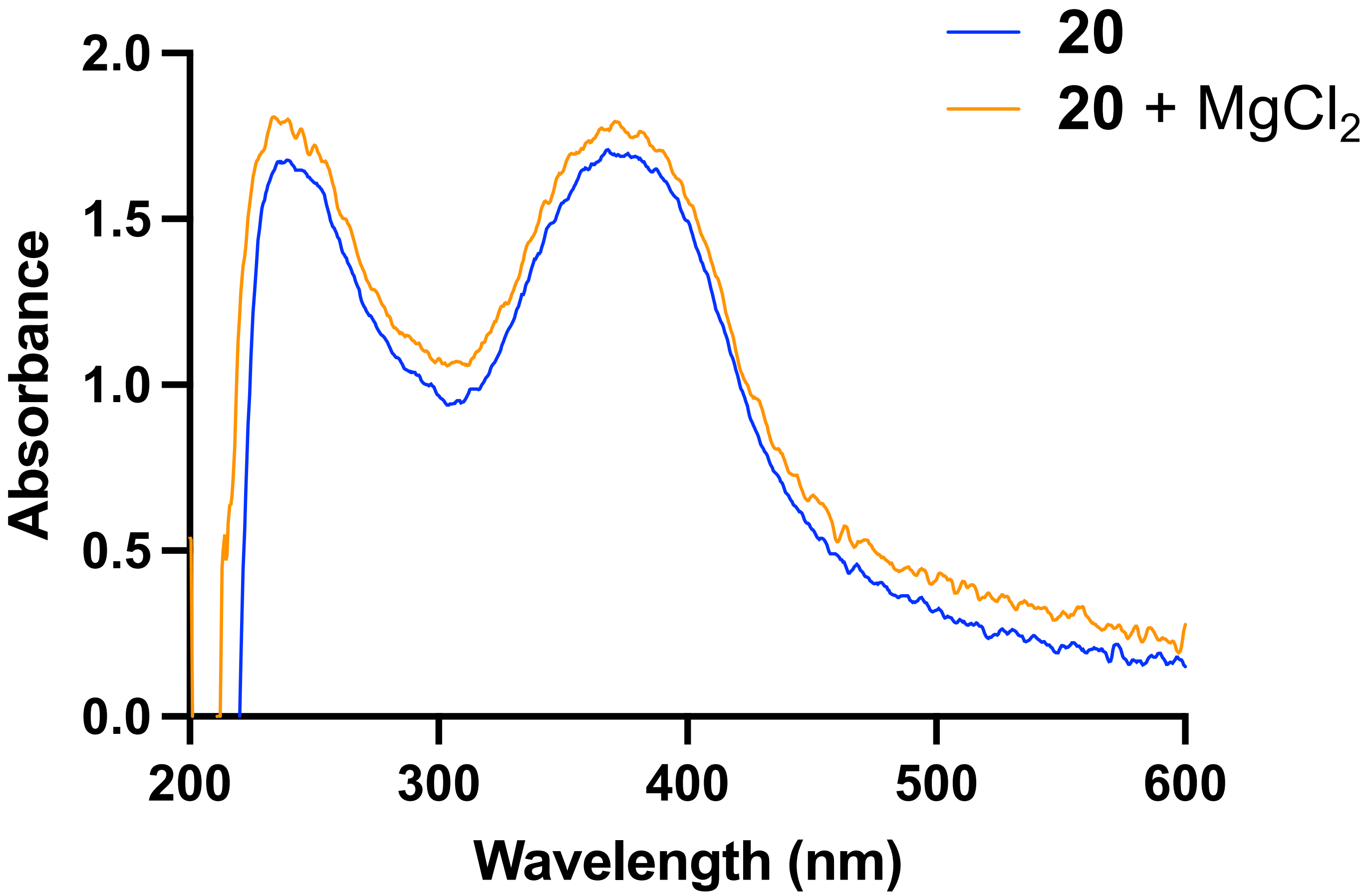

20
20 + MgCl2
2.0
1.5
1.0
0.5
0.0
Absorbance
200
300
400
500
600
Wavelength (nm)

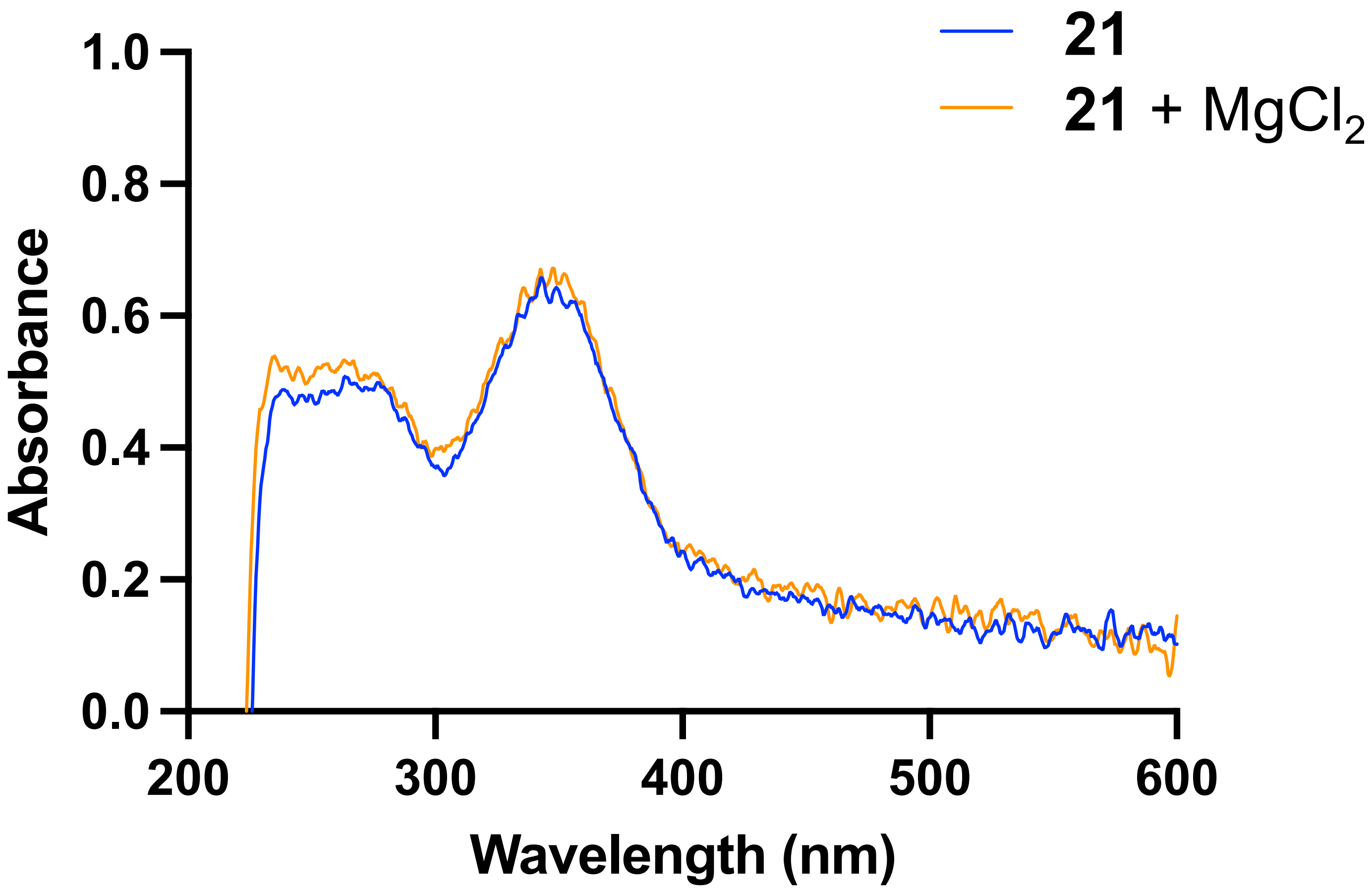

21
21 + MgCl2
1.0
0.8
0.6
0.4
0.2
0.0
Absorbance
200
300
400
500
600
Wavelength (nm)

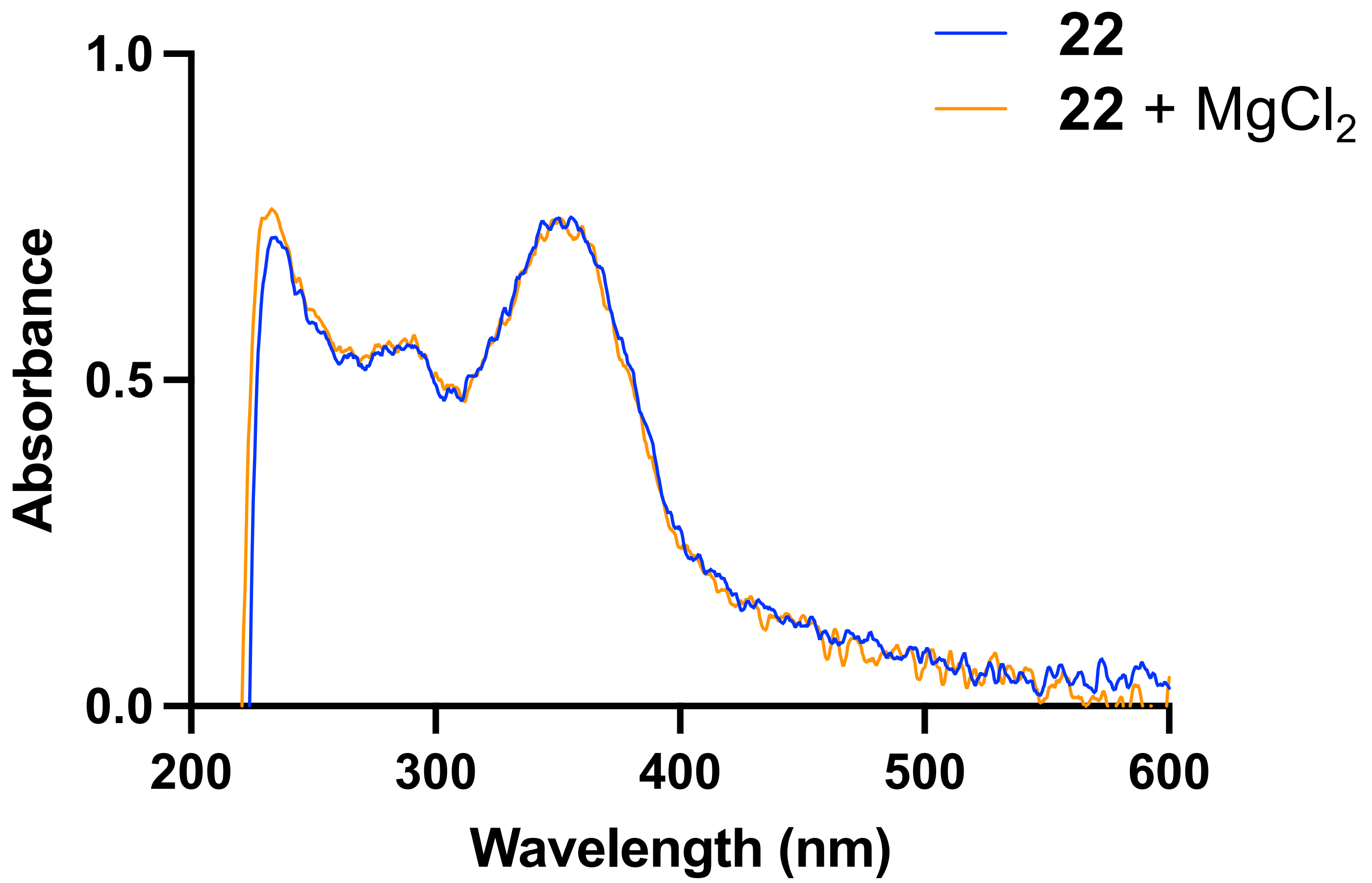
22
22 + MgCl2
Absorbance
1.0
0.5
0.0
200
300
400
500
600
Wavelength (nm)

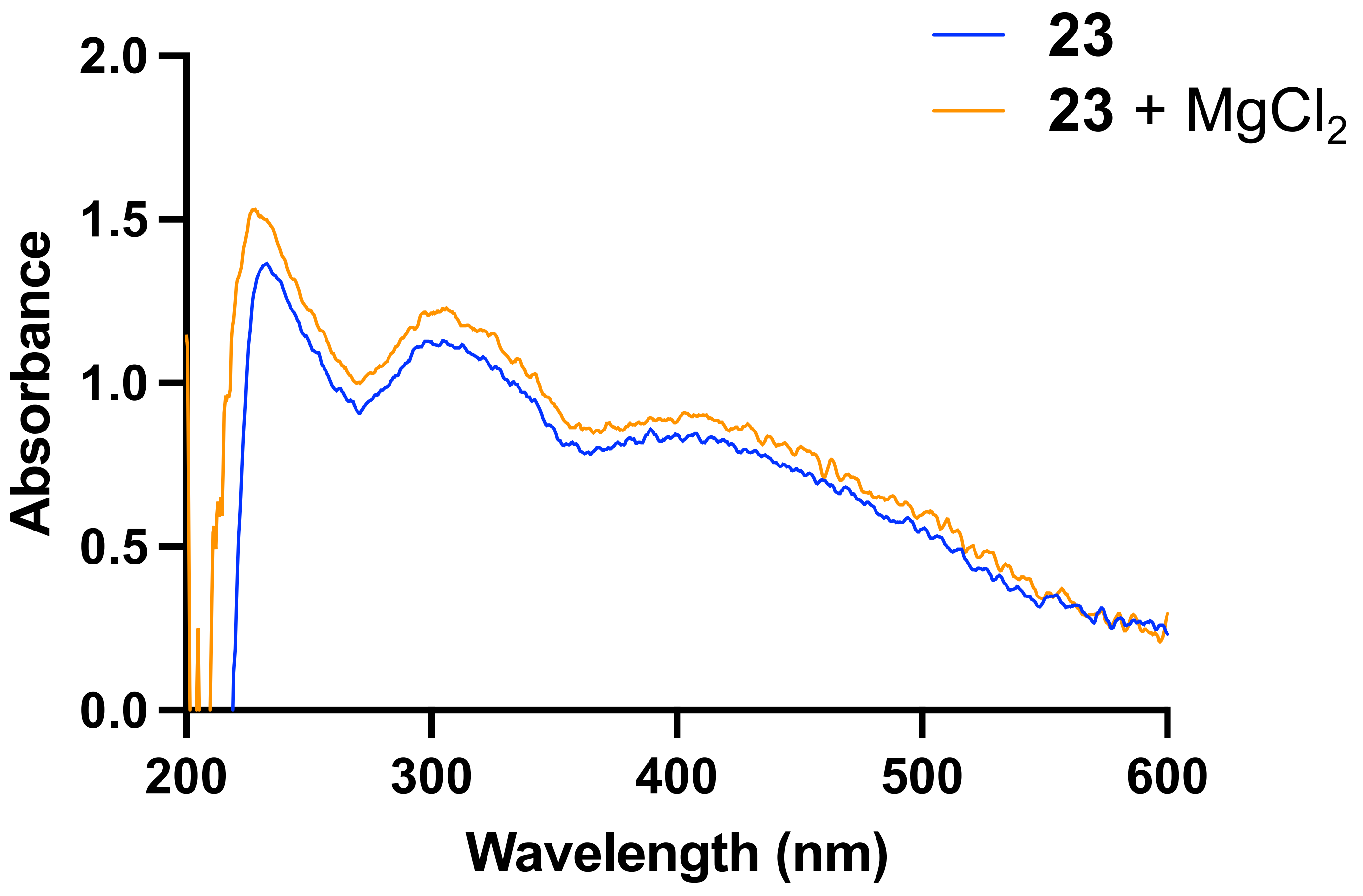
23
23 + MgCl2
Absorbance
2.0
1.5
1.0
0.5
0.0
200
300
400
500
600
Wavelength (nm)

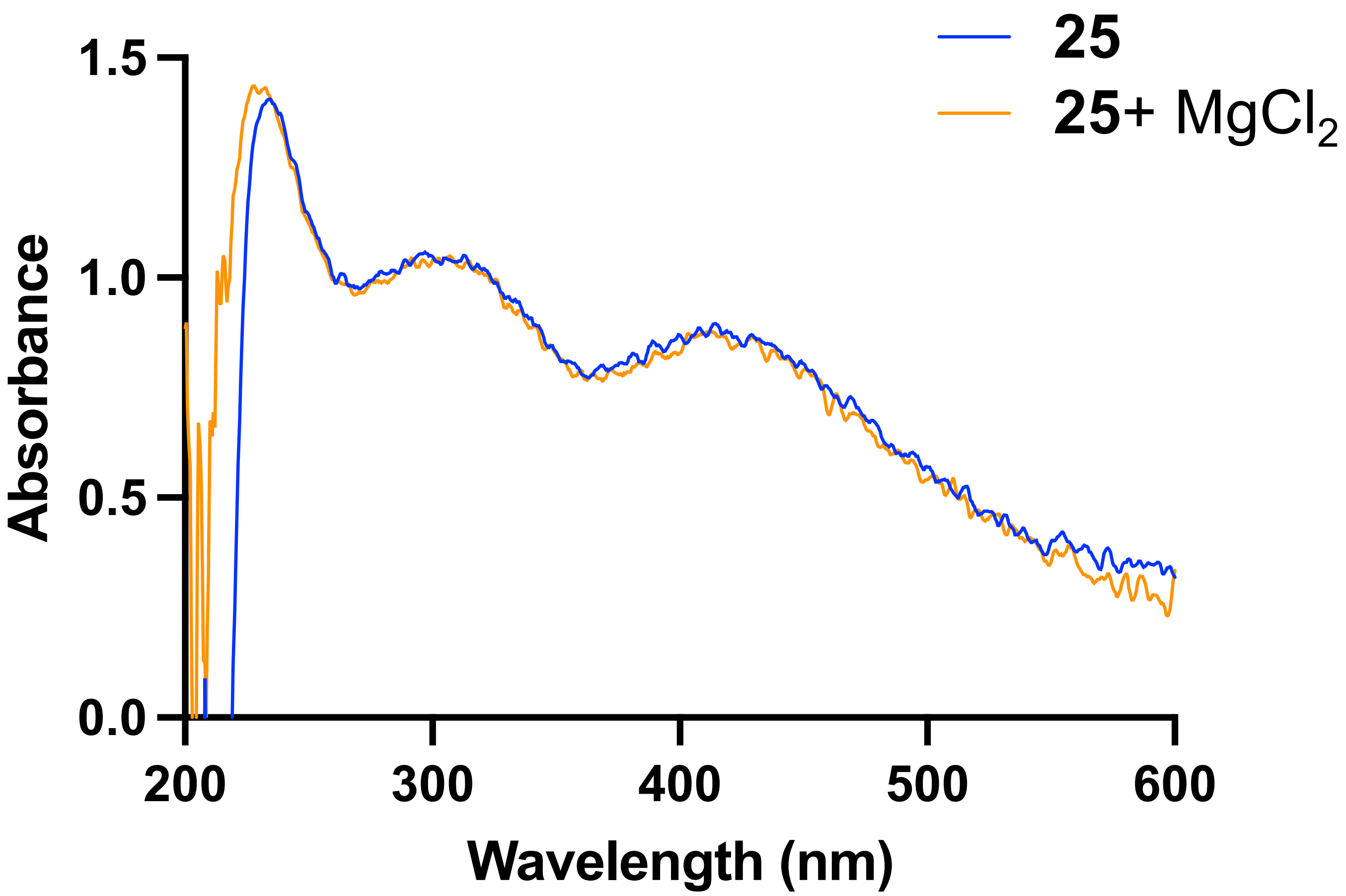
25
25+ MgCl2
Absorbance
1.5
1.0
0.5
0.0
200
300
400
500
600
Wavelength (nm)

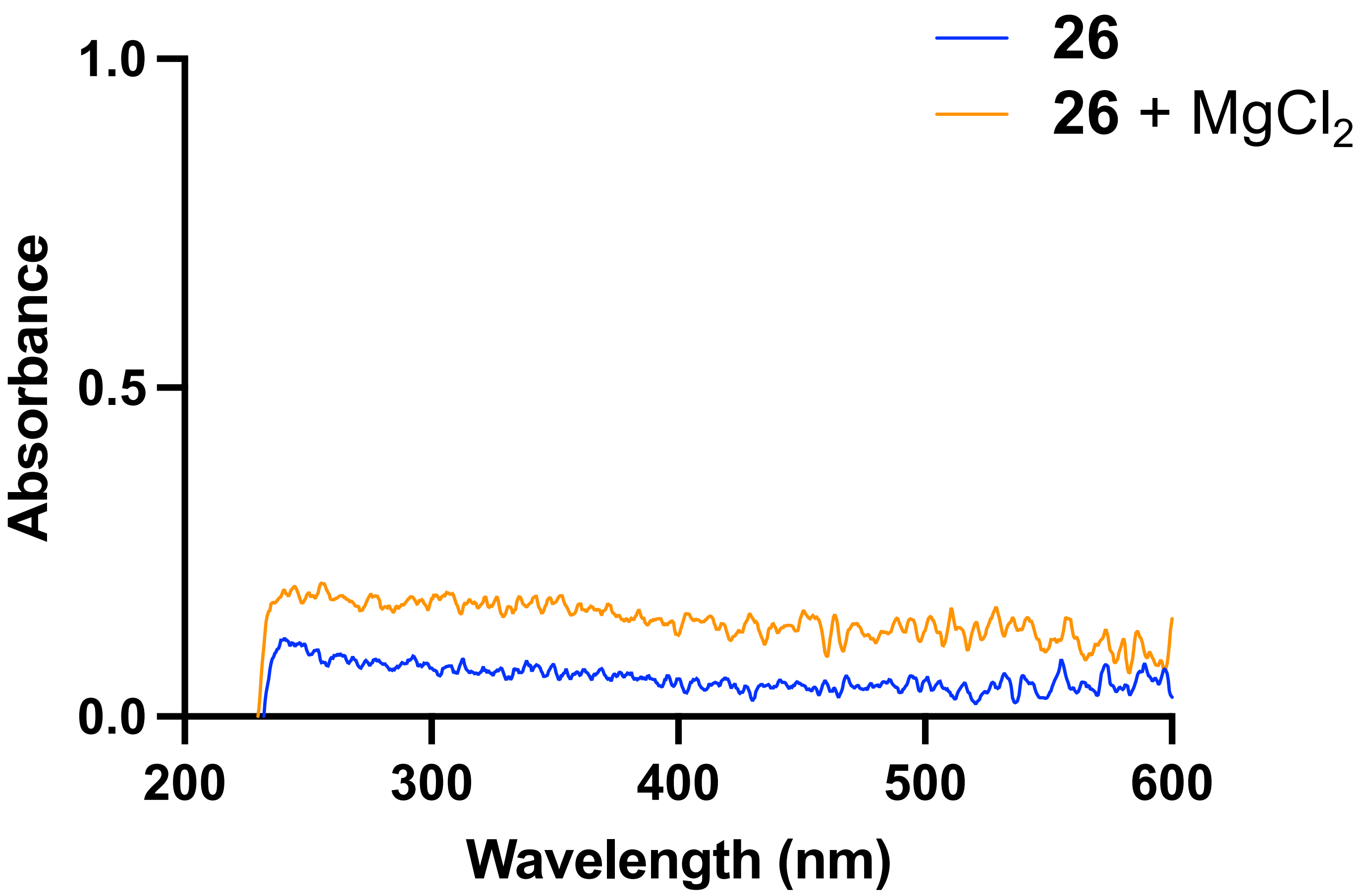
26
26 + MgCl2
Absorbance
1.0
0.5
0.0
200
300
400
500
600
Wavelength (nm)

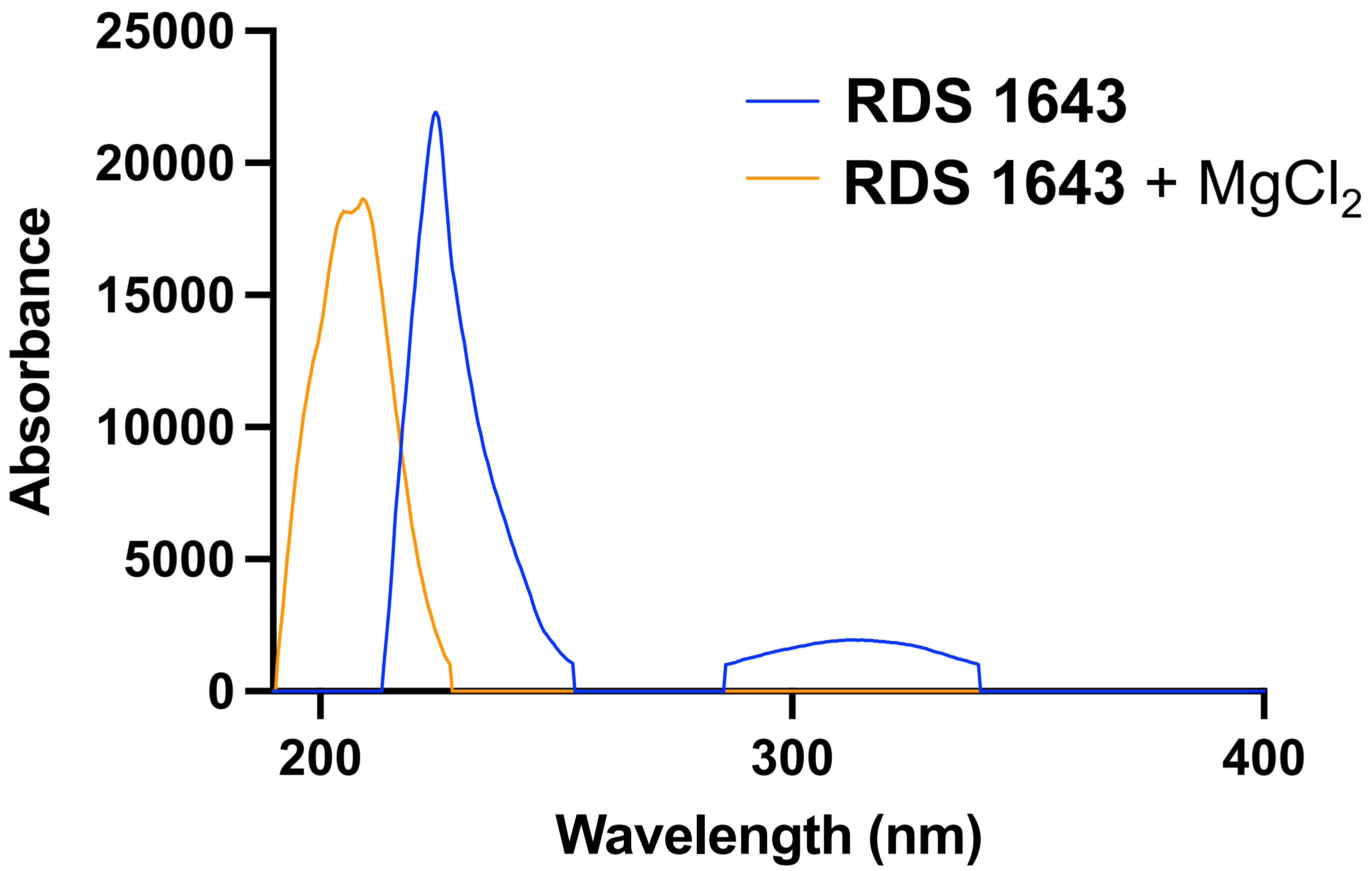
25000
20000
15000
10000
5000
0
Absorbance
200
300
400
Wavelength (nm)
RDS 1643
RDS 1643 + $MgCl_2$
